# Decomposing the role of alpha oscillations during brain maturation

**DOI:** 10.7554/eLife.77571

**Published:** 2022-08-25

**Authors:** Marius Tröndle, Tzvetan Popov, Sabine Dziemian, Nicolas Langer

**Affiliations:** 1 Department of Psychology, University of Zurich, Methods of Plasticity Research Zurich Switzerland; 2 University Research Priority Program (URPP) Dynamic of Healthy Aging Zurich Switzerland; 3 Neuroscience Center Zurich (ZNZ), University of Zurich & ETH Zurich Zurich Switzerland; Uni­ver­sité de Paris France; Carnegie Mellon University United States

**Keywords:** EEG, 1/f, alpha, brain development, thalamocortical connectivity, DTI, Human

## Abstract

Childhood and adolescence are critical stages of the human lifespan, in which fundamental neural reorganizational processes take place. A substantial body of literature investigated accompanying neurophysiological changes, focusing on the most dominant feature of the human EEG signal: the alpha oscillation. Recent developments in EEG signal-processing show that conventional measures of alpha power are confounded by various factors and need to be decomposed into periodic and aperiodic components, which represent distinct underlying brain mechanisms. It is therefore unclear how each part of the signal changes during brain maturation. Using multivariate Bayesian generalized linear models, we examined aperiodic and periodic parameters of alpha activity in the largest openly available pediatric dataset (N=2529, age 5-22 years) and replicated these findings in a preregistered analysis of an independent validation sample (N=369, age 6-22 years). First, the welldocumented age-related decrease in total alpha power was replicated. However, when controlling for the aperiodic signal component, our findings provided strong evidence for an age-related increase in the aperiodic-adjusted alpha power. As reported in previous studies, also relative alpha power revealed a maturational increase, yet indicating an underestimation of the underlying relationship between periodic alpha power and brain maturation. The aperiodic intercept and slope decreased with increasing age and were highly correlated with total alpha power. Consequently, earlier interpretations on age-related changes of total alpha power need to be reconsidered, as elimination of active synapses rather links to decreases in the aperiodic intercept. Instead, analyses of diffusion tensor imaging data indicate that the maturational increase in aperiodic-adjusted alpha power is related to increased thalamocortical connectivity. Functionally, our results suggest that increased thalamic control of cortical alpha power is linked to improved attentional performance during brain maturation.

## Introduction

Childhood and adolescence are critical stages of the human lifespan, in which the brain undergoes various and complex micro- and macroscopic changes (Giedd et al., 1999; Lebel et al., 2008). The typical emergence of mental illnesses during childhood and adolescence (Kessler et al., 2005) further indicates fundamental maturational reorganization. It is therefore particularly important to understand these maturational changes in brain structure and function, which are accompanied by neurophysiological changes. A substantial body of literature has focused on investigating these neurophysiological changes with electroencephalography (EEG) (for reviews, see Anderson and Perone, 2018; Segalowitz et al., 2010). Cognitive functions such as attention and memory, which undergo critical changes during maturation, have frequently been associated with EEG alpha activity (Foxe and Snyder, 2011; Klimesch, 1997; Klimesch, 2012; Niedermeyer, 1999). This lead to the notion that developmental changes in alpha activity reveals mechanisms of cortical manifestations of cognitive function. Indeed, numerous studies have investigated developmental changes in this alpha oscillation and reported evidence of an increase in the individual alpha frequency (IAF) at around 7–14 years of age (Cragg et al., 2011; Díaz de León et al., 1988; Klimesch, 1999; Lindsley, 1939; Marcuse et al., 2008; Niedermeyer, 1999; Somsen et al., 1997). However, the evidence is less clear about the amplitude of this alpha oscillation, termed alpha power. Absolute power was found to decrease with increasing age in some studies (Díaz de León et al., 1988; Gasser et al., 1988; Harmony et al., 1995; Lindsley, 1939; Whitford et al., 2007) but not in others (Clarke et al., 2001; Somsen et al., 1997). A potential confound is the utilization of fixed-frequency boundaries (e.g. 8–13 Hz), which neglects the slowing of the IAF during development. For instance, peak frequency in childhood is around 6 Hz but increases to 10 Hz in adolescents. Hence, age-related power decreases are underestimated when the slower alpha oscillation of younger children is not properly captured by predefined frequency limits, which leads to lower power values. Consequently, individualized alpha frequency bands need to be extracted, which are centered on the individual IAF of each subject (see also Donoghue et al., 2021 for simulations visualizing confounding effects of the peak frequency on band power). In addition, thickening of the skull and other maturational processes that are unrelated to changes in neural activity manifest as changes in overall neural power. Thus, they pose a crucial confound in interpreting the relationship between alpha power and age. To overcome this latter limitation, studies have examined alpha power as relative to the overall power of the spectrum, relative alpha power, which has yielded more consistent results indicating an increase of alpha power with increasing age (Clarke et al., 2001; Cragg et al., 2011; Díaz de León et al., 1988; Harmony et al., 1995; John et al., 1980; Somsen et al., 1997). However, relative power measures of different frequency bands are by definition highly interdependent, as the frequency band of interest is normalized by the power of all the other frequency bands measured (Gómez et al., 2017; Somsen et al., 1997). For example, power changes in other frequency bands, such as the theta band, manifest as changes in relative alpha power, even though the true oscillatory alpha power may remain stable (see Appendix 1—figure 1A). Additionally, non-oscillatory changes in the power spectrum introduce confounds in the analysis of relative band power measures (see simulated example in Appendix 1—figure 1B). This was previously observed by simulations in previous studies showing that non-oscillatory changes affect both relative band power measures Donoghue et al., 2021 and band power ratio measures (Donoghue et al., 2020a). Therefore, the extent to which these earlier works on alpha power and brain maturation are in effect confounded by changes in other frequency bands, by non-oscillatory variations, or by a slowing of the IAF remains unclear. To address this question, true alpha oscillatory power needs to be separated from other, non-oscillatory signal components.

Recent methodological developments have provided a means by which this separation can be achieved. These new approaches decompose measured power into periodic and aperiodic signal components (see Figure 4; Donoghue et al., 2020b; Hughes et al., 2012a; Wen and Liu, 2016). The aperiodic signal (i.e. ‘1/f signal’) is characterized by its intercept and slope, as its amplitude decreases with higher frequencies f. The aperiodic signal contains important physiological information (see He, 2014 for a comprehensive review of the functional significance and potential generative mechanisms of aperiodic activity). More specifically, the aperiodic slope has been linked to the synchronicity of activity in the underlying neural population (Miller et al., 2009; Usher et al., 1995) and its balance between excitatory and inhibitory activity (Gao et al., 2017). Importantly, the aperiodic slope is also modulated by task performance and sensory stimulation (e.g. He, 2014). Conversely, the aperiodic intercept has been linked to general spiking activity (Voytek and Knight, 2015). Overall, the aperiodic signal needs to be considered during the analysis of spectral power rather than measuring power relative to the absolute zero (e.g. Donoghue et al., 2020b). Applying this new approach of spectral decomposition allows to extract an aperiodic-adjusted measure of alpha power, which is independent of oscillatory activity in other frequency bands and changes in overall power and aperiodic activity.

Recent studies adopted this methodology and found age-related changes in the aperiodic signal (i.e., decreased intercept and flattened slope) during childhood and adolescence (Cellier et al., 2021; Hill et al., 2022) and from childhood to middle age (Donoghue et al., 2020a; He et al., 2019). These results further pointed out the importance of considering the aperiodic signal in the investigation of alpha power during brain maturation. However, it remains largely unknown how aperiodic-adjusted alpha power evolves during this critical phase of life. The few studies performed so far have not found any significant association between aperiodic-adjusted alpha power during childhood and adolescence (Cellier et al., 2021; Hill et al., 2022) and from childhood to middle age (He et al., 2019). Due to comparatively small sample sizes in these studies, it remains unclear whether the aperiodic-adjusted alpha power truly remains stable in this period of life or whether too little statistical power was provided to detect changes in this newly emerging measure of alpha power. Furthermore, conventional measures of total and relative alpha power were either not reported (Cellier et al., 2021; Hill et al., 2022), or did not show any relation to age (He et al., 2019). Hence, comparisons and integration of these results with the large body of literature investigating maturational changes in total and relative alpha power remain limited.

To overcome limitations of previous studies, we analyzed the currently largest openly available pediatric EEG data set (N=2529), comprising children adolescents and young adults aged between 5 and 22 years (Alexander et al., 2017) and validated the results in a second, preregistered analysis (https://osf.io/7uwy2) of a dataset consisting of 369 children, adolescents, and young adults aged between 6 and 22 years.

Based on animal studies investigating cortical and subcortical neural generators of alpha activity in adult animals (Bishop, 1936; Lopes da Silva, 1991; Steriade et al., 1990), it is generally assumed that the thalamus and thalamocortical interactions strongly modulate cortical alpha activity. It can thus be hypothesized that changes in the alpha oscillation in the maturing brain are driven by structural changes of the thalamus and thalamocortical connectivity. However, to the best of our knowledge, no study investigated the relationship between anatomical maturation of the thalamus and its connectivity and alpha oscillatory power during childhood and adolescence. To close this gap, we further examined how thalamic structural changes relate to the observed changes in the different measures of alpha power. We employed magnet resonance images (MRI) to operationalize changes in the thalamus in terms of thalamic volume, and diffusion tensor imaging (DTI) to estimate thalamocortical connectivity by white matter integrity of the thalamic radiation. This was done in a subsample of the larger main dataset, for which magnet resonance images (MRI) and diffusion tensor imaging (DTI) data were additionally available.

Taken together, the present study aims to delineate the role of alpha oscillations during brain maturation by investigating conventional and newly emerging alpha oscillatory parameters, aperiodic signal components (see Table 7) and its underlying anatomical basis in a large sample of children, adolescents and young adults. Our first goal is to replicate previous literature that reported an age-related increase of the IAF, increased relative alpha power and decreased total alpha power during brain maturation. However, we hypothesize that the relation of alpha band power and brain maturation is no longer present when adjusting alpha power for the aperiodic signal component and for the age-related increase of the IAF. Additionally, based on previous literature we expect a decrease in the aperiodic intercept and a flattening of the aperiodic slope during brain maturation. Finally, as the aperiodic-adjusted individualized alpha power is unbiased by changes in other frequency bands and the aperiodic signal component, and therefore is likely to provide the most accurate reflection of the true oscillatory activity, we hypothesize that this measure shows a significant association with thalamic anatomical measures.

## Results

### Main analysis

**Figure 1.**
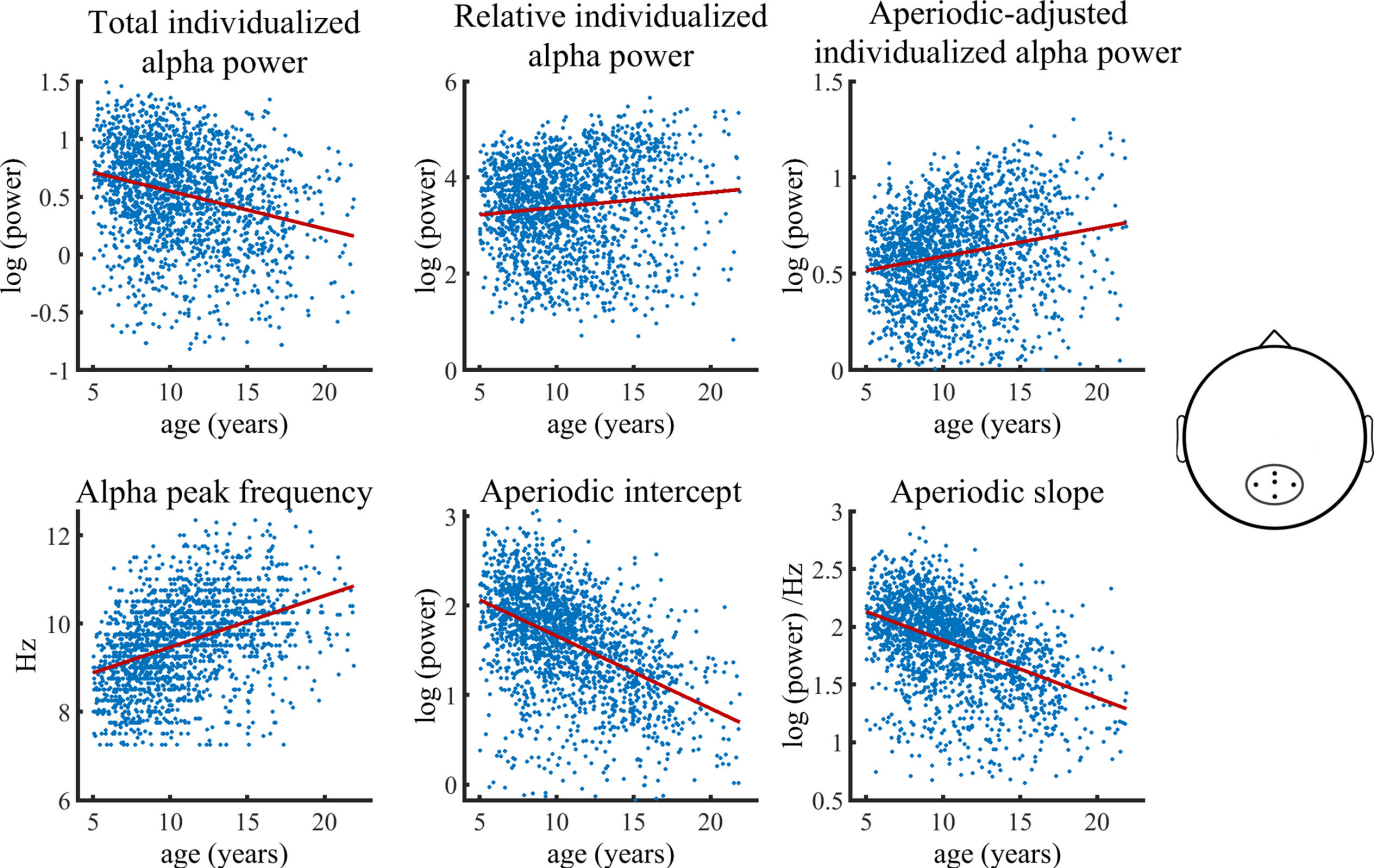
Visualization of data of the main HBN sample used in the Bayesian regression model. Solid lines represent fitted regression lines. The schematic head on the right indicates the location of the electrode cluster from which data was aggregated. The results revealed a decrease in total individualized alpha power with increasing age. Importantly, this relationship inverts when individualized alpha power is adjusted for the aperiodic signal, which then shows an age-related increase in power. Furthermore, relative individualized alpha power exhibits a positive relationship to brain maturation. An age-related increase of the IAF and a decrease of the aperiodic intercept and slope are also indicated (bottom row). Figure 1—source data 1.Numerical data for each scatterplot displayed in Figure 1.

The relation of age to the various parameters estimated in the Bayesian regression model are shown in Figure 1.

**Figure 1—figure supplement 1.**
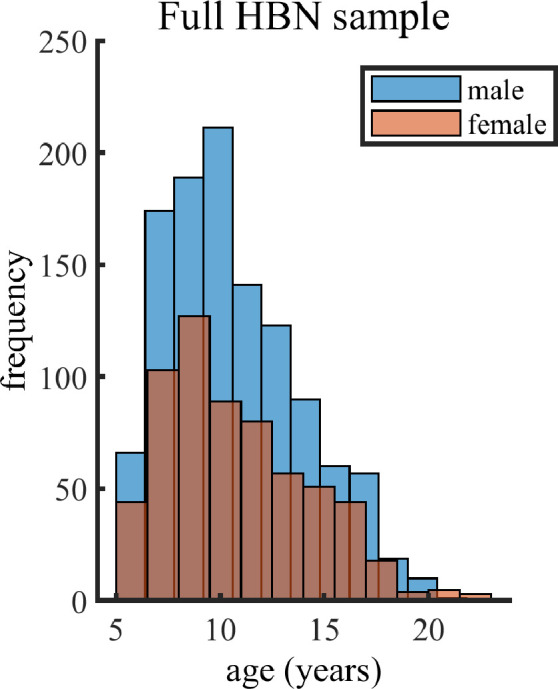
Distribution of age and gender in the final included sample plotted in Figure 1 and used for the statistical analyses.

**Figure 1—figure supplement 2.**
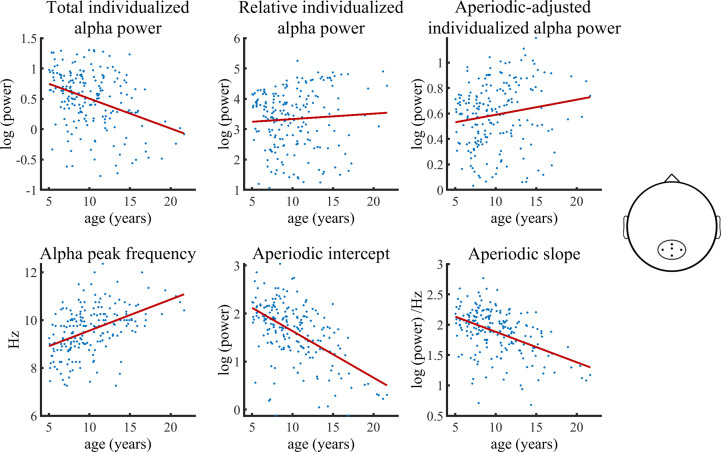
Visualization of data of the HBN subsample without any given diagnosis used in the Bayesian regression models. Solid lines represent fitted regression lines. The schematic head (right) indicates the location of the electrode cluster from which data was aggregated.

The Bayesian regression model provided significant evidence for a reduction of total individualized alpha power during brain maturation (*b=*–0.31*, CI =* [-0.38,–0.24]). In contrast, a significant increase with increasing age was observed for aperiodic-adjusted alpha power (*b=*0.23*, CI =* [0.16, 0.30]). Relative alpha power also showed a significant increase with increasing age (*b=*0.14*, CI =* [0.06, 0.22]). The model further provided significant evidence for an age-related decrease in both the aperiodic slope (*b=*–0.44*, CI =* [-0.51,–0.38]) and the aperiodic intercept (*b=*–0.54*, CI =* [-0.61,–0.48]). Females showed overall less power in total, relative, and aperiodic-adjusted individualized alpha and a lower aperiodic intercept in slope. The statistical models controlled for the heterogeneity of the full sample by adding a categorical diagnosis variable. The categorical diagnosis predictor did not show any significant effect on any of the outcomes. Table 1 summarizes the results of the Bayesian regression model. A control analysis, in which we divided the ADHD diagnosis into two sub diagnoses of inattentive type and combined type, did not change any results (see Supplementary file 2). Most importantly, a control analysis including only the subsample without any given diagnosis, showed results highly consistent with those of the full sample (see Figure 1—figure supplement 2 and Supplementary file 2A).

**Table 1.** Bayesian regression model results of full sample with categorical diagnosis predictor. Table 1—source data 1.Numerical values of the statistical values displayed in Table 1.

Outcome	β_predictor_ [CI]				
	age	gender	diagnosis: ADHD	diagnosis: Other	age*gender
alpha peak frequency	0.42 [0.34 0.49]	–0.08 [–0.15 –0.02]	–0.09 [–0.19 0.01]	–0.07 [–0.17 0.04]	–0.04 [–0.15 0.08]
total individualized alpha power	–0.31 [–0.38 –0.24]	–0.37 [–0.43 –0.31]	0.01 [–0.09 0.10]	0.01 [–0.09 0.12]	0.13 [0.01 0.25]
Relative individualized alpha power	0.14 [0.06 0.22]	–0.35 [–0.41 –0.28]	–0.01 [–0.11 0.10]	0.01 [–0.10 0.12]	–0.05 [–0.17 0.07]
aperiodic-adjusted individualized alpha power	0.23 [0.16 0.30]	–0.39 [–0.45 –0.33]	–0.04 [–0.14 0.05]	–0.02 [–0.13 0.08]	–0.06 [–0.17 0.06]
aperiodic intercept	–0.54 [–0.61 –0.48]	–0.37 [–0.42 –0.32]	–0.02 [–0.10 0.07]	–0.02 [–0.10 0.08]	0.08 [–0.02 0.19]
aperiodic slope	–0.44 [–0.51 –0.38]	–0.39 [–0.44 –0.33]	–0.04 [–0.12 0.05]	–0.03 [–0.12 0.06]	–0.05 [–0.15 0.06]

Note: Credible Interval (CI)=99.17%.

Analyses of canonically defined alpha power measures demonstrated a significant negative age effect on total canonical alpha power (*b*=–0.10, *CI* = [-0.17,–0.12]). Its effect size was significantly smaller than that of the age effect on total individualized alpha power (*CI* is non-overlapping: [-0.38,–0.24]). Relative and aperiodic-adjusted canonical alpha power showed consistent significant age-related increases. See Supplementary file 2C for detailed results on canonical alpha power.

**Figure 2.**
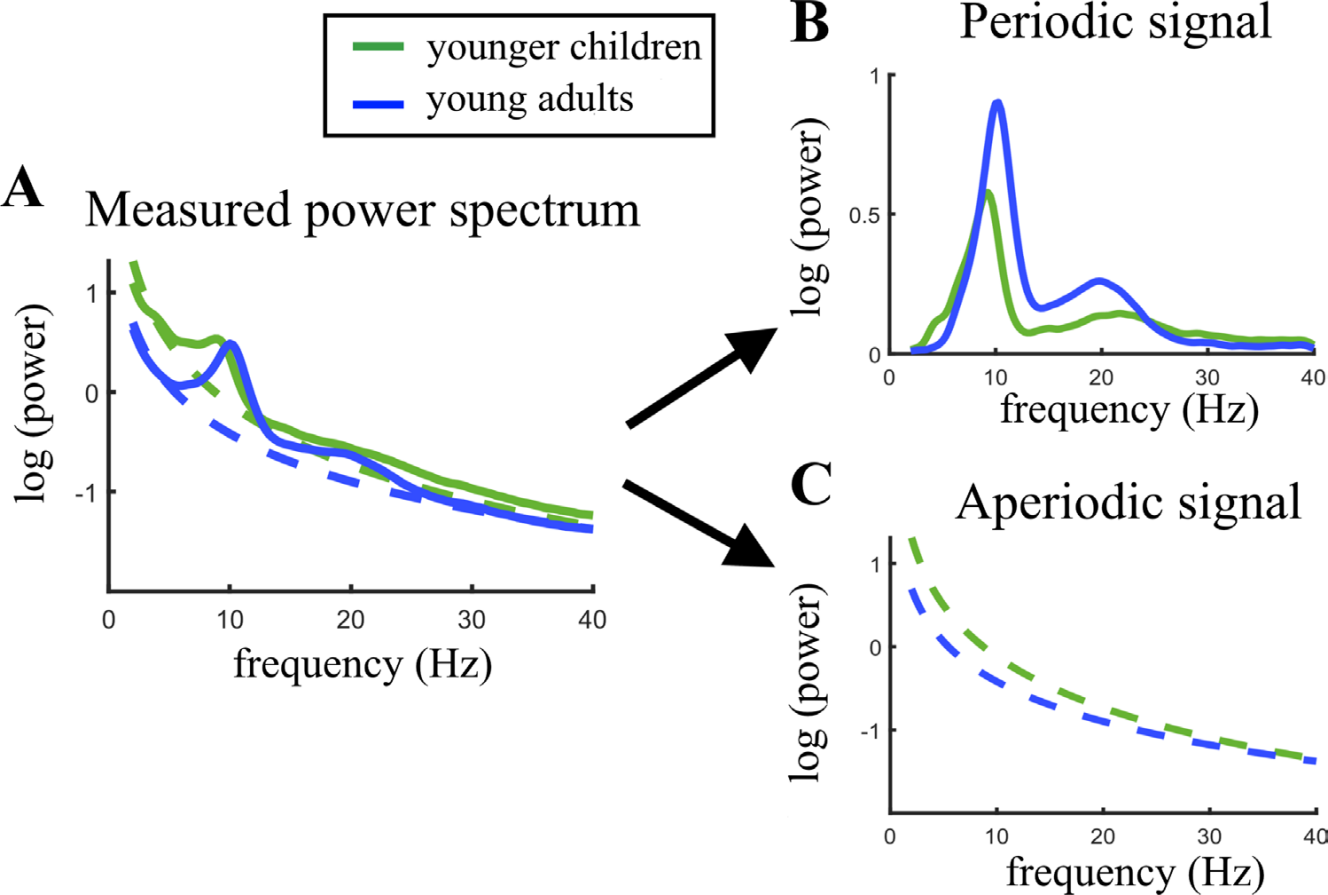
Visualization of age-related changes during brain maturation in (**A**) the measured power spectrum (i.e., total power spectrum), (**B**) the periodic (i.e. aperiodic-adjusted) power spectrum, and (**C**) the aperiodic signal. Younger children represent the 20% youngest children in the sample, young adults the 20% oldest participants. This split of the sample was only done for visualization purposes and not used in any statistical analysis. Figure 2—source data 1.Numerical averaged data of the periodic and aperiodic signal of both age groups.

Figure 2 illustrates the age-related changes in the PSD during brain maturation. For visualization purposes, and in contrast to the statistical model, which used a continuous age variable, the grand averages for the youngest 20% (5.04–7.75 years) and oldest 20% (13.68–21.90 years) participants were plotted across the parieto-occipital electrodes. Figure 2A indicates an age-related decrease in alpha power in the total power spectrum, caused by lower total power values in young adults in the lower frequency range of the alpha band (here ~7–10 Hz). Figure 2B visualizes the altered group differences when adjusting the power spectrum for the aperiodic signal. Here, an increase is observed in aperiodic-adjusted alpha power. The periodic signal shows a decreased intercept and flattened slope in age (Figure 2C).

To investigate the inherent associations between the different measures of alpha power and aperiodic activity, we further conducted a post hoc correlational analysis. This analysis aimed to illuminate the differences and similarities between the three measures of alpha power and the potential confounding effects of the aperiodic signal. The two aperiodic indices, intercept and slope, were highly interlinked, and both exhibited high correlations with total individualized alpha power. At the same time, both aperiodic-adjusted and relative individualized alpha power indicated a considerably weaker association with the aperiodic indices. Furthermore, whereas relative and aperiodic-adjusted alpha power were highly related, this association was weaker between relative and total alpha power and aperiodic-adjusted and total alpha power. Table 2 summarizes the result of the correlational analysis.

**Table 2.** Pearson correlation coefficients between the measures of alpha power, aperiodic intercept and slope and age. Table 2—source data 1.Numerical values of the correlations displayed in Table 2.

	Total individualized alpha power	Relative individualized alpha power	Aperiodic-adjusted individualized alpha power	Aperiodic intercept	Aperiodic slope
Total individualized alpha power		0.66	0.64	0.84	0.64
Relative individualized alpha power			0.88	0.34	0.35
Aperiodic- adjusted individualized alpha power				0.34	0.36
Aperiodic intercept					0.89

### Validation analyses

Results of the validation analyses were mostly consistent with those obtained from the main HBN sample. In terms of age effects, all results were replicated; however, the age-related decrease of relative individualized alpha power failed to reach significance. Here, neither gender nor the ADHD diagnosis showed any significant effect on the outcome measures. Table 3 summarizes the results of the statistical models using uninformative Cauchy priors.

**Table 3.** Bayesian regression model results of the full validation sample with categorical ADHD predictor. Table 3—source data 1.Numerical values of the statistical values displayed in Table 3.

Outcome	β_predictor_ [CI]			
	Age	Gender	ADHD diagnosis	Age*gender
Alpha peak frequency	0.34 [0.15 0.53]	–0.05 [–0.21 0.11]	–0.04 [–0.20 0.12]	0.04 [–0.25 0.33]
Total individualized alpha power	–0.44 [–0.61 –0.27]	–0.05 [–0.19 0.11]	0.04 [–0.11 0.19]	–0.07 [–0.33 0.30]
Relative individualized alpha power	0.20 [0.00 0.39]	0.05 [–0.12 0.22]	–0.02 [–0.18 0.15]	–0.10 [–0.40 0.20]
Aperiodic-adjusted individualized alpha power	0.33 [0.14 0.52]	–0.02 [–0.18 0.13]	–0.04 [–0.21 0.12]	–0.06 [–0.35 0.24]
Aperiodic intercept	–0.76 [–0.88 –0.65]	–0.10 [–0.19 0.00]	0.00 [–0.10 0.09]	–0.07 [–0.25 0.11]
Aperiodic slope	–0.60 [–0.77 –0.45]	–0.08 [–0.21 0.05]	–0.06 [–0.19 0.06]	–0.13 [–0.36 0.10]

Note: CI = 98.97% Credible Interval, gender variable is dummy coded: 1=female, 0=male.

**Figure 3.**
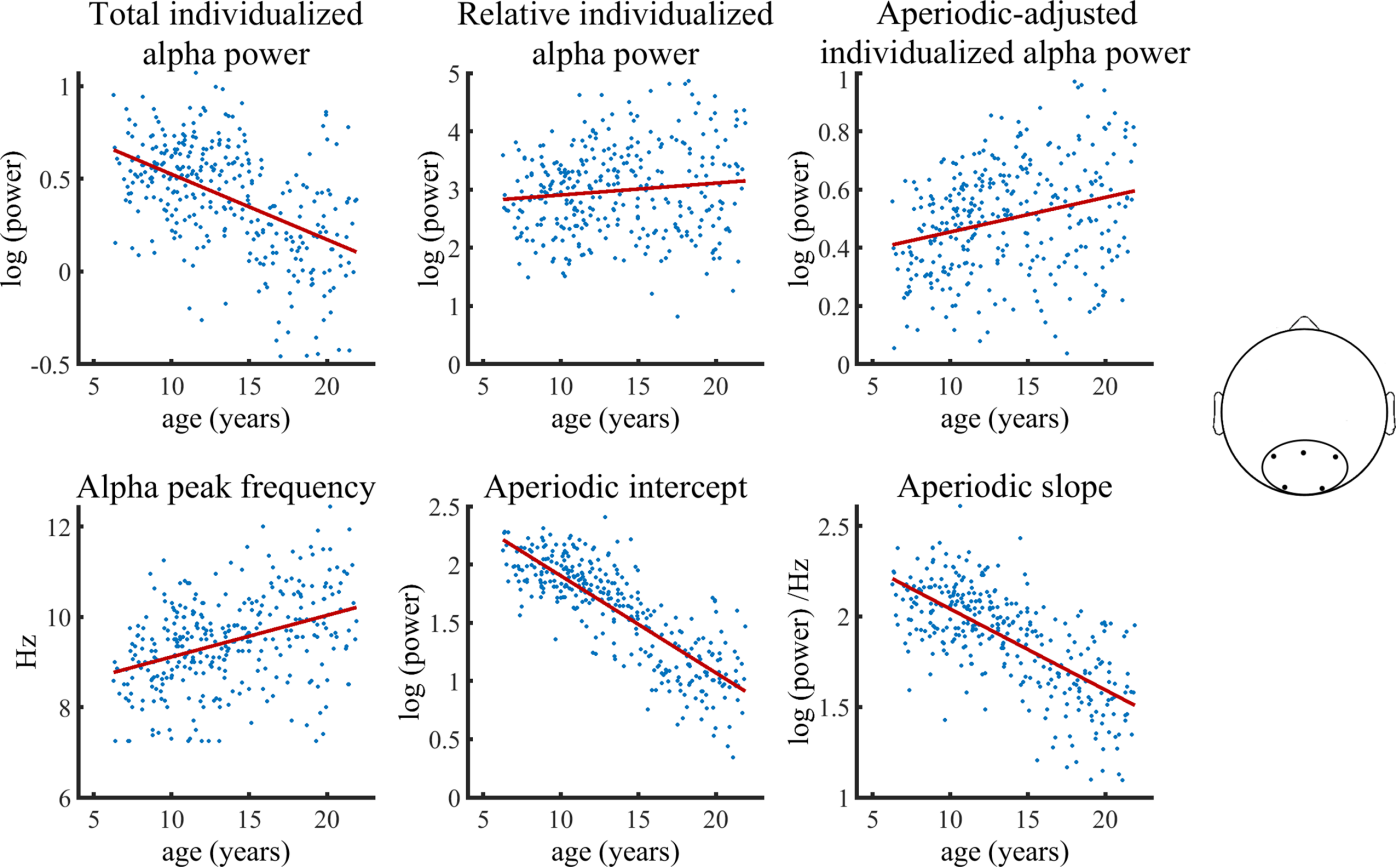
Visualization of data of the validation sample used in the Bayesian regression model. Solid lines represent fitted regression lines. The schematic head on the right indicates the location of the electrode cluster from which data was aggregated. Figure 3—source data 1.Numerical data for each scatterplot displayed in Figure 3.

Figure 3 visualizes age trajectories of the outcome measures in the full validation subsample.

**Figure 3—figure supplement 1.**
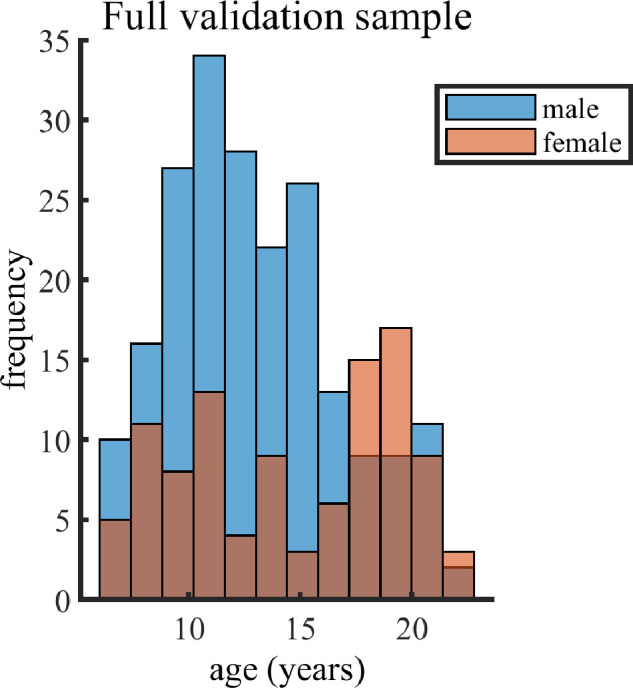
Distribution of age and gender in the final included validation sample plotted in Figure 3 and used for the statistical analyses.

**Figure 3—figure supplement 2.**
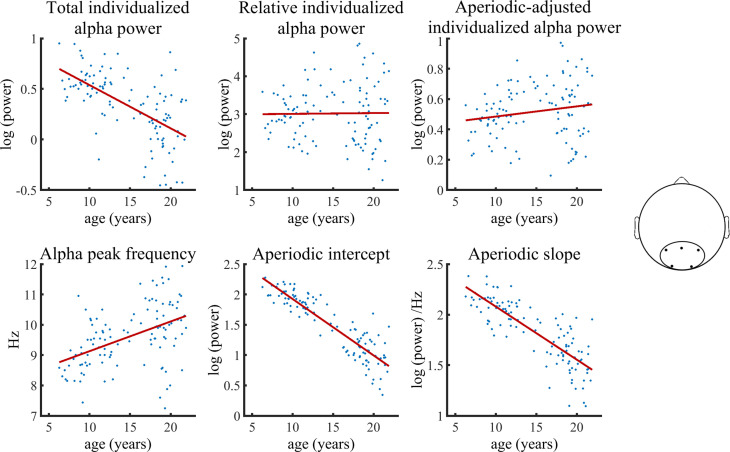
Visualization of data of the healthy validation subsample used in the Bayesian regression models. Solid lines represent fitted regression lines. The schematic head (right) indicates the location of the electrode cluster from which data was aggregated.

Importantly, the control analysis, which included only the healthy subsample, showed results highly consistent with those of the full sample (see Figure 3—figure supplement 2 and Supplementary file 3A).

As an additional analysis, we applied Bayesian sequential updating to accumulate evidence across the different datasets. This procedure allows to increase statistical power and to produce more generalizable research outcome, which is less specific to each of the investigated samples. Therefore, informative priors extracted from the statistical results of the main sample were applied. Age effects on the six outcome variables in this analysis showed evidence consistent with the results obtained from the separate data sets. Gender showed significant effects on the three measures of alpha power and the aperiodic signal components here, although no gender effects were observable in the validation analyses using uninformative Cauchy priors. Because of the large difference between sample sizes (N_main_ = 1770, N_validation_ = 310), the extracted priors had a strong influence on the results; thus, it remains unclear how these gender effects generalize to other datasets. Clinical diagnosis of ADHD did not show any significant influence in the main and the validation analysis, however, in this analysis combining evidence across the two dataset, there was a significant negative relationship between ADHD diagnosis and aperiodic-adjusted alpha power, but not with total or relative alpha power. See Supplementary file 3B for detailed results.

### Relation of alpha power and the aperiodic signal to anatomical thalamic measures

Table 4 summarizes the results of the analysis on the relation of white matter integrity of the left/right thalamic radiation (derived from DTI data), thalamus volume and total intracranial volume (both extracted from and T1-weighted MRI data) with the different measures of alpha power and aperiodic activity. No relation was observed between thalamus volume and any outcome variable. Both the left and right thalamic radiation showed significant associations with aperiodic-adjusted individualized alpha power, which did not reach significance level with total individualized alpha power. Relative alpha power showed similar results to those observed with aperiodic-adjusted alpha power. No significant associations were found between the aperiodic intercept and slope and the thalamic radiations.

**Table 4.** Summary table of the Bayesian regression model results on the influence of anatomical thalamic measures on the three different measures of alpha power (total, relative and aperiodic-adjusted) and on aperiodic signal components. Table 4—source data 1.Numerical values of the statistical values displayed in Table 4.

Outcome	β_predictor_ [CI]					
	Left thalamic radiation	Right thalamic radiation	Thalamus volume	Total intracranial volume	Age	Gender
Total individualized alpha power	0.06 [–0.02 0.14]	0.07 [–0.01 0.15]	0.03 [–0.23 0.29]	–0.01 [–0.14 0.13]	–0.23 [–0.31 –0.15]	–0.35 [–0.43 –0.27]
Aperiodic-adjusted individualized alpha power	0.15 [0.07 0.23]	0.15 [0.07 0.23]	0.07 [–0.19 0.33]	–0.04 [–0.18 0.10]	0.22 [0.14 0.30]	–0.39 [–0.47 –0.31]
Relative alpha power	0.11 [0.04 0.20]	0.14 [ 0.06 0.22]	0.00 [–0.27 0.27]	–0.01 [–0.15 0.13]	0.14 [0.07 0.22]	–0.35 [–0.43 –0.27]
Aperiodic intercept	0.04 [–0.04 0.10]	0.02 [–0.05 0.09]	–0.01 [–0.24 0.23]	0.03 [–0.09 0.15]	–0.51 [–0.58 –0.44]	–0.35 [–0.42 –0.28]
Aperiodic slope	0.07 [0.00 0.14]	0.03 [ –0.03 0.11]	–0.11 [–0.34 0.13]	–0.03 [–0.13 0.17]	–0.48 [–0.55 –0.41]	–0.39 [–0.45 –0.31]

Note: CI = 98.52% Credible interval, gender variable is dummy coded: 1=female, 0=male. Credible intervals and β estimates of the covariates (total intracranial volume, age, and gender) showed minor deviations across the three models (see 4.4.3, models 4, 5 & 6), due to non-deterministic sampling in the Bayesian model estimation. Therefore, here they were averaged across the three models.

### Relation to Flanker task scores

Post hoc analyses were performed to investigate the relation between the different measures of alpha power and performance in a visual spatial attention task: the Flanker task of the National Institutes of Health Toolbox Cognition Battery (Gershon et al., 2013). Both relative and aperiodic-adjusted individualized alpha power showed a significant positive association with task performance while the effects of age and gender were controlled for. The effect of Flanker task performance on total alpha power showed a considerably smaller effect size (standardized β dropped by a factor of 1.7 compared to aperiodic-adjusted alpha power) and failed to reach significance level when adjusting for multiple comparisons. Table 5 summarizes the results of the linear models.

**Table 5.** Linear models determining the influence of Flanker total task scores on total, relative, and aperiodic-adjusted individualized alpha power. Table 5—source data 1.Numerical values of the statistical values displayed in Table 5.

Outcome	standardized β_predictor_ (standard error)		
	Flanker total score	Age	Gender
Aperiodic-adjusted individualized alpha power	0.073 (0.022), p=0.001	0.23 (0.022), p<0.001	–0.75 (0.045), p<0.001
Total individualized alpha power	0.044 (0.022), p=0.046	–0.23 (0.022), p<0.001	–0.74 (0.045), p<0.001
Relative individualized alpha power	0.068 (0.023), p=0.002	0.14 (0.023), p<0.001	–0.68 (0.047), p<0.001

Adjusted significance level: P=0.0264.

## Discussion

This study investigated the role of alpha oscillations during brain maturation. Our results replicated the well-documented finding of an increasing IAF in this developmental phase of life from childhood to adolescence. The main aim of the study was to delineate the developmental trajectory of the power of the alpha oscillation by considering changes in the individual alpha frequency and aperiodic signal components in a large sample size. A significant decrease of total individualized alpha power was observed with increasing age across samples. However, when correcting for the aperiodic signal, the results changed considerably. The aperiodic-adjusted individualized alpha power increased significantly from childhood to adolescence, which is consistent with the results obtained from relative alpha power in the present study and in previous literature (Clarke et al., 2001; Cragg et al., 2011; Díaz de León et al., 1988; Harmony et al., 1995; John et al., 1980; Somsen et al., 1997). The aperiodic signal showed a decreased intercept during brain maturation and a flattened slope. Results were largely consistent across the subsample of the HBN dataset without any given diagnosis, the full HBN dataset, and the validation analyses. However, in the validation dataset and the HBN subsample without any given diagnoses, aperiodic-adjusted but not relative alpha power showed significant age-related increases, indicating a risk of false-negative results when investigating relative alpha power in brain maturation. The covariates of psychiatric diagnoses did not show any significant influences on oscillatory alpha or aperiodic signal parameters in the two datasets. Only in the Bayesian sequential updating analysis, combining both datasets, ADHD was associated with decreased aperiodic-adjusted alpha power. Future research is needed to investigate possible relations between psychiatric diseases and periodic and aperiodic EEG signal components in more detail, which is beyond the scope of the present report. Gender effects on alpha power and aperiodic signal components were found in the HBN dataset; however, these effects did not generalize on the validation sample. Importantly, when relating alpha power measures to anatomical measures derived from DTI, only aperiodic-adjusted and relative alpha power showed a significant relation to the white matter integrity of the thalamic radiations, but total alpha power did not.

### Age related changes in IAF and total canonical alpha power

The often-replicated increase of IAF from childhood to adolescence was also observed here. Higher IAF was previously linked to better sensorimotor abilities (Mierau et al., 2016) and increased memory performance (Klimesch, 1999). It was hypothesized that the mechanism underlying the increased memory performance is an increase in the speed of information processing, which was directly linked to the IAF by various studies investigating reaction time paradigms (Jin et al., 2006; e.g. Klimesch et al., 1996; Surwillo, 1961; Surwillo, 1961). This matches the findings of generally increased speed of information processing from childhood to adolescence (Kail, 2000). Thus, the increasing IAF observed during brain maturation may represent the neurophysiological correlate of increased speed of information processing. It has further been hypothesized that this is related to developmental increases in myelination and axon size (Cragg et al., 2011; Segalowitz et al., 2010).

In terms of alpha power analyses, the IAF increase induces a bias when alpha power is investigated with the canonical fixed-frequency bands, as was predominantly done in earlier investigations Clarke et al., 2001; Díaz de León et al., 1988; Gasser et al., 1988; Harmony et al., 1995; Klimesch, 2012; Somsen et al., 1997; Whitford et al., 2007. Although some of these studies, Díaz de León et al., 1988; Gasser et al., 1988; Harmony et al., 1995; Whitford et al., 2007, found an age-related decrease in this total canonical alpha power measure, the present study only demonstrated a significant relationship between canonical alpha power and age in the large samples consisting of both healthy children, adolescents and young adults and those diagnosed with ADHD or other psychiatric disorders. In contrast, the age effect on the alpha power adjusted for changes in IAF (i.e. total individualized alpha power) was significant across all datasets. It showed an effect size three times larger than that of the total canonical alpha power. Thus, the developmental decrease on total alpha power may be underestimated when using canonical frequency bands. This is because the slower alpha oscillation of younger children is not properly captured by predefined band limits and thus yields a lower alpha power value. Thus, we highly recommend refraining from using canonical fixed-frequency bands when investigating age-related changes in alpha power.

Even after accounting for the age-related changes in IAF, interpreting changes in total individualized alpha power still remains problematic. As noted earlier (e.g. Benninger et al., 1984; John et al., 1980; Matthis et al., 1980), a relative alpha power measure should be preferred over total alpha power, as the latter is more dependent on non-neurophysiological changes such as skull thickness and skin conductivity and is consequently less reliable (John et al., 1980).

### Relative vs. aperiodic-adjusted individualized alpha power

Relative individualized alpha power exhibited a positive relation to age in the full HBN sample, in contrast to the decrease in total individualized alpha power relative to the developmental trajectory. Thus, when overall changes in the power spectrum are taken into account, alpha power increased with increasing age. However, normalizing alpha power by the power of all the other frequency bands measured poses problems for the interpretation of results. Post hoc simulations indicate that changes in power in other frequency bands (see Appendix 1—figure 1A) induce changes in relative alpha power even when true oscillatory alpha power is kept constant. Furthermore, changes in the aperiodic signal induce a confound in the relative alpha power measure (see Appendix 1—figure 1B). This is further supported by simulations performed by Donoghue et al., 2020a and Donoghue et al., 2021. Consequently, the increase in relative alpha power observed with increasing age needs to be interpreted with caution, as changes in other frequency bands and in the aperiodic signal can potentially bias this finding. Our study confirmed an age-related decrease of the aperiodic intercept and a flattening of the aperiodic slope. Hence, because these changes in the aperiodic signal could induce changes in relative alpha power even though the true oscillatory pattern remains stable, this relative measure is no conclusive indicator of a true age-related increase in alpha power.

Nonetheless, the present findings on the empirical data demonstrated that both aperiodic-adjusted alpha power and relative alpha power show similar results with respect to a maturational increase. Thus, our results are in line with previous findings reporting age-related increases in relative alpha power (Clarke et al., 2001; Cragg et al., 2011; Díaz de León et al., 1988; Harmony et al., 1995; John et al., 1980; Somsen et al., 1997). Additionally, the analyses relating alpha power measures to possible neuroanatomical (thalamocortical connectivity) and behavioral (visual attention task performance) correlates yielded very similar significant positive associations with both relative and aperiodic-adjusted alpha power. Supplementary, post hoc correlational analyses (see Table 2) showed highly comparable associations between the aperiodic signal components with relative alpha power (aperiodic intercept: *r*=0.34, aperiodic slope: *r*=0.35) and with aperiodic-adjusted alpha power (aperiodic intercept: *r*=0.34, aperiodic slope: *r*=0.36), hence indicating no distinct confounding effects of either the aperiodic intercept or slope on relative alpha power. Moreover, these analyses showed a high correlation (*r*=0.88) between aperiodic-adjusted and relative alpha power. However, this also indicated still considerable residual unexplained variance (22.6%) between these two measures of alpha power. In fact, Bayesian regression model results demonstrated that maturational changes are underestimated when investigating relative alpha power (*b*=0.14) compared to aperiodic-adjusted alpha power (*b*=0.23), which is also reflected in post hoc performed correlational analyses between age and the two measures of alpha power (*r*_relative alpha power_ = 0.11, *r*_aperiodic-adjusted alpha power_ = 0.21). Importantly, the aperiodic-adjusted individualized alpha power showed consistent significant age-related increases in the main HBN sample, the HBN subsample of children without any given diagnosis, and the validation dataset. Conversely, the relative individualized alpha power only showed a significant association with age in the largest main HBN sample. Therefore, our results indicate that there is a risk of false negative results when investigating relative alpha power changes from childhood to young adulthood in sample sizes commonly used in neurophysiological studies. Hence, the developmental increase on periodic alpha power may be underestimated when using relative alpha power indices, which might be explained by a potential confounding bias of the aperiodic signal components and power in other frequency bands on the relative alpha power (see supplementary simulation studies in Appendix 1). Overall, aperiodic-adjusted alpha power should be preferred over relative alpha power when analyzing developmental trajectories during brain maturation.

### Age-related increase in aperiodic-adjusted individualized alpha power

A significant increase in aperiodic-adjusted individualized alpha power with increasing age was observed across samples. As discussed above, this measure of alpha power most likely reflects the true oscillatory changes in this phase of life, because its extraction is independent of changes in the aperiodic signal and other frequency bands. The separation of periodic and aperiodic activity minimizes confounding factors such as the observed maturational decreases in overall power (i.e. in the aperiodic intercept) or the maturational flattening of the aperiodic slope, which are inseparable in conventional measures of alpha power. This is further supported by post hoc correlational analyses (see Table 2). High correlations were observed between total alpha power and the aperiodic intercept (*r*=0.83) and the aperiodic slope (*r*=0.63), which were considerably smaller in aperiodic-adjusted alpha power (aperiodic intercept: *r*=0.33, aperiodic slope: *r*=0.35). Thus, compared to aperiodic-adjusted alpha power, findings on age-related decreases in total alpha power are likely to be much more strongly driven by changes in the aperiodic signal, in particular by the decrease observed in the aperiodic intercept. Consequently, one could interpret that changes in total alpha power reflect changes in the aperiodic signal rather than periodic alpha power. Although other recent studies using smaller sample sizes and varying age ranges did not find an association of aperiodic-adjusted alpha power and brain maturation (Cellier et al., 2021; He et al., 2019; Hill et al., 2022), the present study provides strong evidence for an age-related increase across two large datasets.

Previous studies on age-related changes of alpha power during brain maturation speculated that decreased total oscillatory power may be due to synaptic pruning processes (e.g. Cragg et al., 2011) and thus reflect decreased spiking activity. The increase in aperiodic-adjusted alpha power provides new insights into these interpretations: Decomposing the neural power spectra rather indicates that these processes relate to changes in the aperiodic signals intercept (see discussion section ‘Maturational changes in aperiodic signal components’). Another speculative hypothesis was that the observed developmental changes in the alpha band reflect structural changes in the thalamus and thalamocortical connectivity (Cragg et al., 2011; Whitford et al., 2007). However, this specific link was not formally investigated, but was based on early work on animal models, which explored adult cortical and subcortical neural generators of alpha activity. These models suggested that, in the adult animal brain, interacting thalamocortical loops may primarily be involved in the generation of the alpha rhythm (Bishop, 1936). Subsequent animal studies provided further evidence that the neural generators of alpha oscillations may be the occipital cortex, under strong guidance of the visual thalamus (Lopes da Silva, 1991; Lopes da Silva et al., 1973). So far, to the best of our knowledge, no studies were conducted to investigate if human maturational changes in the different measures of alpha power relate to anatomical changes in the thalamus and its connectivity. To close this missing link, we tested whether the resting-state alpha oscillatory power measures extracted here are related to anatomical measures of thalamic volume and thalamocortical connectivity, here measured by white matter integrity of the thalamic radiation. A significant positive relationship between white matter integrity of the thalamic radiation and aperiodic-adjusted individualized alpha power was observed. Thus, the current study provides evidence that the increase in aperiodic-adjusted alpha power (and relative alpha power) during brain maturation is related to increases in thalamocortical connectivity. The statistical models also indicated that this relation is not purely based on co-maturation of alpha power and structural connectivity, because significant relations were found between these measures after controlling for effects of age and the total intracranial volume. Total alpha power did not show any significant relation to these anatomical measures; hence, it is further indicated that this measure of alpha power is confounded by aperiodic signal components or non-neurophysiological changes during brain maturation. After age and total intracranial volume were controlled for, no association was found between thalamic volume and any of the alpha power measures. It could therefore be speculatively hypothesized that changes in cortical alpha oscillations are not related to anatomical changes in the thalamus. Instead, the increases observed in alpha power may relate to improved connectivity between the neural generators in the thalamus and cortical neural populations.

Alterations in thalamocortical connectivity have been positively linked to attention and working memory performance in aging (Charlton et al., 2010; Hughes et al., 2012b; Ystad et al., 2011) and infancy (Alcauter et al., 2014; Ball et al., 2015). Importantly, these cognitive functions have typically also been associated with the amplitude of alpha oscillation (Bazanova and Vernon, 2014; Foxe and Snyder, 2011; Klimesch, 1997; Klimesch, 1999; Klimesch, 2012) and are well known to improve during brain maturation. Furthermore, a highly influential simultaneous EEG and fMRI study (Laufs et al., 2003) hypothesized, based on correlations between cortical EEG and the blood oxygen level-dependent (BOLD) signal in frontoparietal regions, that adult resting state alpha power is linked to internally focused attention. These findings indicate that maturational increases of thalamic regulation of cortical alpha power may relate to improvements in attentional performance. In fact, post hoc analyses supported this hypothesis by providing evidence that oscillatory alpha power is linked to performance in visual attention tasks, assessed by performance in the Flanker task. While age, gender, and handedness were controlled for, relative and aperiodic-adjusted individualized alpha power showed significant positive relations to the attentional performance when adjusting for multiple statistical comparisons, but total individualized alpha power did not. Taken together, contrary to findings based on total alpha power, aperiodic-adjusted individualized alpha power increased during brain maturation and seems likely to be related to increased thalamocortical connectivity. Functionally, this increase in alpha power is linked to improvements in attentional performance.

### Maturational changes in aperiodic signal components

The decreases in the aperiodic intercept and slope with increasing age are in line with previous observations in different age groups (Cellier et al., 2021; Donoghue et al., 2020a; He et al., 2019). One explanation for this decrease of the aperiodic intercept may lie within maturational changes that are unrelated to neural mechanisms. As previous studies on EEG changes during brain maturation have suggested (e.g., Dustman et al., 1999), the thickening of the skull increases its resistance. This increased resistance could lead to the observed decrease in broadband EEG power, here reflected in a decrease in the aperiodic intercept. However, this maturational effect was also found in broadband power (Gómez et al., 2017) and the aperiodic intercept (He et al., 2019) in studies using magnetoencephalography (MEG), which is not affected by skull thickness.

Alternatively, as previous studies have shown that the aperiodic intercept is related to the overall spiking activity of the underlying neural population (Miller et al., 2009), the decrease observed in the aperiodic intercept may reflect a reduced parieto-occipital spiking activity during brain maturation. This observation may be related to the finding that as much as 40% of synapses in the striate cortex are eliminated during brain development (Huttenlocher and de Courten, 1987). This is further supported by Whitford et al., 2007, who found a reduction of gray matter volume in parietal cortex co-occurring with a decrease in broadband EEG power during brain development, suggesting that this may reflect synaptic pruning processes. Yet, more research is needed to delineate possible mechanisms underlying this age-related decrease observed in the aperiodic intercept. An alternative explanation for the finding of a decreased intercept needs to be considered: As pointed out by He et al., 2019, a maturational flattening of the aperiodic signal imposes a decrease in the intercept due to the high correlation between the aperiodic intercept and slope (also observed in the main HBN dataset, *r*=0.89, see Table 2). To estimate whether the observed decrease of the aperiodic intercept is larger than expected by the rotation of the aperiodic slope requires estimation of not only the decrease of the aperiodic slope, but also the frequency at which the aperiodic signal rotates. Future research is needed to provide means by which this estimation can be achieved, considering also interindividual differences in the rotation frequency.

The flattening of the aperiodic signal in this age range may also be reflected in the commonly observed age-related decrease of power in low frequencies accompanied by an increase in power in higher frequencies (Cragg et al., 2011; Whitford et al., 2007). This phenomenon was speculated to be related to the elimination of synapses or changes in white matter structure (Segalowitz et al., 2010; Whitford et al., 2007); however, no significant relation of the aperiodic slope with white matter integrity of the thalamic radiation was found in the analyses performed here. An additional post hoc analysis also indicated no relation between the aperiodic signal parameters and global white matter integrity (see Supplementary file 4). Yet, considering a shift of the aperiodic slope in the interpretation of this shift in power of frequency bands could provide additional insights into this little-understood result. Flattening of the aperiodic slope has been linked to changes in the excitation–inhibition ratio of the neural population (Gao et al., 2017), particularly to an increase in local excitatory feedback. This shift in the excitation–inhibition ratio causes temporally decorrelated spikes and thus increases in neural noise (Voytek and Knight, 2015). This is supported by earlier studies relating a decreased aperiodic slope to more asynchronous activation patterns in neural populations (Miller et al., 2009; Usher et al., 1995). Thus, the flattened slope observed here might reflect increases in neural noise during brain development (McIntosh, 2010). This may seem contradictory, as aging research has linked an increase in neural noise to age-related cognitive decline from adulthood to old age (Voytek et al., 2015). However, increasing neural noise might rather have beneficial effects in the earlier processes of brain maturation. Reviewing maturational studies that used EEG and fMRI, McIntosh, 2010 concluded that the maturing brain develops from a deterministic to a more stochastic system in which the increased neural noise leads to enhancement of functional network potential.

### Limitations

A limitation of the present study is the composition of the samples investigated, as they contain a large proportion of children, adolescents, and young adults in whom psychiatric disorders were diagnosed. Consequently, the samples are not representative of the general population in this age range. This may present a confound to the analysis of age trajectories of alpha power and the aperiodic signal, because psychiatric disorders have previously been linked to differences in resting state EEG band power (for a comprehensive review, see Newson and Thiagarajan, 2018) and the aperiodic slope (e.g. Robertson et al., 2019). However, control analyses using only healthy subsamples showed very similar results to analyses of the full sample. Additionally, the main and the validation analysis controlled for possible confounding effects by adding a categorical diagnosis variable as an additional predictor. No significant associations were found between clinical diagnoses and either oscillatory or aperiodic signal components within either dataset.

### Conclusions

This study has demonstrated the relevance of taking the alpha peak frequency and aperiodic signal components into account when assessing age-related changes in spectral power during brain maturation. Our results indicate that there is significant variation of aperiodic activity during childhood and adolescence, which poses a confound to earlier work. Moreover, canonically defined frequency bands render age-related changes in IAF and power inseparable. Accounting for these confounding factors, and using the largest openly available pediatric sample, the present report demonstrates that aperiodic-adjusted alpha power increases during brain maturation. Although previous recent studies did not find any relation between aperiodic-adjusted alpha power and age, the here applied robust statistical models provide strong evidence for an age related increase in a large dataset and across several control analyses. Moreover, the results on aperiodic-adjusted individualized alpha power and the aperiodic signal intercept and slope were confirmed in an independent preregistered validation study, indicating that these spectral EEG measures are robust markers of the maturing brain.

In addition, the spectral decomposition into periodic and aperiodic signal components provides explanations for the previous ambiguous results of studies investigating total or relative alpha power changes during brain maturation. The present study provides evidence that the maturational decreases in total alpha power are conflated by the maturational changes of the aperiodic signal components. Thus, previous interpretations that total alpha power may be related to the elimination of active synapses need to be reconsidered, as the decomposition of the neural power spectrum reveals that these processes may rather relate to decreases of the aperiodic intercept. Furthermore, the current report provides partial support of previous literature on age-related increases in relative alpha power, as these effects could only be replicated in the large dataset, but not in the smaller samples. Consequently, aperiodic-adjusted alpha power should be preferred over relative alpha power, as the latter measure underestimated age-related changes of true periodic alpha power and therefore yielded a risk of false negative results. Instead, aperiodic-adjusted alpha power increases with increasing age, and likely reflects the development of thalamocortical connectivity. Functionally, these maturational changes may relate to increased attentional performance.

## Materials and methods

All analysis code described below is available at https://osf.io/4nzyk/. This repository further contains all extracted EEG features and demographics which were used in the statistical models.

### Datasets

For the main study, 2529 resting-state EEG datasets were obtained from the Human Brain Network (HBN) project (Alexander et al., 2017). The HBN project by the Child Mind Institute is an ongoing initiative that aims to generate a freely available biobank of multimodal datasets of children, adolescents and young adults aged 5–22 years. All participants undergo a variety of assessments. For the current study, we retrieved the Edinburgh handedness inventory (EHI, Oldfield, 1971), the Wechsler Intelligence Scale for Children-V (WISC-V, Wechsler, 2003) for children and adolescents aged 6–17 or the Wechsler Adult Intelligence Scale (WAIS-IV, Wechsler, 2008) for young adults older than 18 years, demographic data on age and gender, and clinical diagnoses. The clinical diagnoses are assessed by licensed clinicians who apply the DSM-5-based Schedule for Affective Disorders and Schizophrenia - Children’s version (KSADS) psychiatric interview (Kaufman et al., 1997). Additionally, when available, the corresponding MRI and DTI datasets were obtained (N=851). Of the 2529 downloaded EEG datasets, 78 were not further processed because the EEG files were either corrupt or the recording length was not sufficient (i.e. the file size was smaller than 30 megabytes). Of the 2451 remaining EEG datasets, 174 could not be used because demographic data was missing. Applying the objective and reproducible exclusion criteria described below yielded a final sample size of 1770 subjects. See Appendix 2—figure 1 for a detailed flow chart of all exclusion criteria and the resulting sample size. Table 6 provides an overview of the final sample characteristics. Because the large proportion of children, adolescents and young adults with a diagnosed psychiatric disorder within the HBN sample might bias findings on general brain maturation, we conducted additional sensitivity analyses by subsampling this sample, including only subjects without any given diagnosis. This additional subsample consisted of 190 subjects.

**Table 6.** Characteristics of the final sample. Table 6—source data 1.Numerical values of the demographics displayed in Table 6.

Characteristic	HBN subsample without any given diagnosis	Full HBN sample	Healthy validation subsample	Full validation sample
Sample size	190	1770	108	310
Female	86	632	62	103
Male	104	1138	46	207
Mean age in years (sd)	10.07 (3.39)	10.81 (3.44)	12.97 (3.75)	13.51 (4.16)
Mean IQ (sd)	106.16 (15.24)	98.86 (16.55)	107.95 (13.13)	103.37 (14.77)

For the preregistered validation study, a second dataset was employed which had previously been collected in a multicentric study. This dataset consisted of 369 children, adolescents and young adults aged 6–22 years and contained both participants with ADHD and a healthy subsample (see Table 6). In this sample, IQ was measured by CFT 1 R (Weiß, 2011) for children below the age of 9 years, CFT 20 R part I (Weiß, 2011) for children and adolescents between 9 and 16 years, and WMT-2 for adolescents and young adults older than 16 years (Forman et al., 2006). Table 6 summarizes sample characteristics of the final sample (see Figure 1—figure supplement 1 for a visualization of the distribution of age and gender in the HBN sample, and Figure 3—figure supplement 1 for the validation sample).

### Main study

#### Experimental setup and procedure

The participants of the HBN sample were comfortably seated in a chair in a sound‐shielded room at a distance of 70 cm from a 17-inch CRT monitor (SONY Trinitron Multiscan G220, display dimensions 330×240 mm, resolution 800×600 pixels, vertical refresh rate of 100 Hz). The room was equipped with a chinrest to minimize head movements. Subjects were informed that EEG would be recorded while they rested with their eyes alternately open or closed. Instructions for the tasks were presented on the computer screen, and a research assistant answered questions from the participant from the adjacent control room through an intercom. Compliance with the task instructions was confirmed through a live video-feed to the control room. The task procedure was that participants rested with their eyes open for 20s (a total of 1min 40s), followed by 40s (a total of 3min 20s) with their eyes closed, repeated five times. Prerecorded verbal instructions automatically informed the participants when to open or close their eyes via loudspeakers. Participants were asked to maintain a fixed gaze on the fixation cross throughout EO blocks. The total duration of the EEG recording was 5min. The alternating order of EO and EC was designed to avoid fatigue and maintain vigilance. The duration of EC blocks was twice as long as EO blocks because eyes-closed data is more robust and contains fewer artifacts. This protocol has been used in various previous studies (Langer et al., 2012; Langer et al., 2013).

#### Electroencephalography recording and preprocessing

The EEG was recorded at a sampling rate of 500 Hz using a high‐density 128‐channel EEG Geodesic Netamps system (Electrical Geodesics, Eugene, Oregon). The recording reference was at Cz, the vertex of the head, and impedances were kept below 40 kΩ.

All analyses were performed using MATLAB 2018b (The MathWorks, Inc, Natick, Massachusetts, United States). EEG data was automatically preprocessed using the current version (2.4.3) of the MATLAB toolbox Automagic (Pedroni et al., 2019). Our pipeline consisted of the following steps. First, bad channels were detected by the algorithms implemented in the clean_rawdata eeglab plugin (http://sccn.ucsd.edu/wiki/Plugin_list_process). A channel was defined as a bad electrode when data recorded by that electrode was correlated at less than 0.85 with an estimate based on other channels. Furthermore, a channel was defined as bad if it had more line noise relative to its signal than all other channels (four standard deviations). Finally, a channel was considered bad if it had a longer flat-line than 5 s. These bad channels were automatically removed and later interpolated using a spherical spline interpolation (EEGLAB function eeg_interp.m). The interpolation was later performed as a final step before the automatic quality assessment of the EEG files (see below). Next, data was filtered using a high-pass filter (–6 dB cut off: 0.5 Hz). Line noise artifacts were removed by applying Zapline (Cheveigné, 2020), removing seven power line components. Subsequently, independent component analysis (ICA) was performed. Components reflecting artifactual activity were classified by the pretrained classifier ICLabel (Pion-Tonachini et al., 2019). Components which were classified as any class of artifacts, including line noise, channel noise, muscle activity, eye activity, and heart artifacts, with a probability higher than 0.8 were removed from the data. Subsequently, residual bad channels were excluded if their standard deviation exceeded a threshold of 25 μV. Very high transient artifacts (>±100 μV) were excluded from the calculation of the standard deviation of each channel. However, if this resulted in a significant loss of channel data (>25%), the channel was removed from the data. After this, the pipeline automatically assessed the quality of the resulting EEG files based on four criteria: A data file was marked as bad-quality EEG and not included in the analysis if, first, the proportion of high-amplitude data points in the signals (>30 μV) was larger than 0.20; second, more than 20% of time points showed a variance larger than 15 microvolt across channels; third, 30% of the channels showed high variance (>15 μV); and fourth, the ratio of bad channels was higher than 0.3. Finally, 13 of 128 electrodes in the outermost circumference, attached to chin and neck, were excluded from further processing as they capture little brain activity and mainly record muscular activity. Additionally, 10 EOG electrodes were separated from the data and not used for further analysis, yielding a total of 105 EEG electrodes. Data was then referenced to the common average reference.

#### Spectral analysis

Spectral analysis was performed on data from the concatenated five blocks of the eyes-closed condition. Only data from the eyes-closed condition was analyzed, because this data contains fewer artifacts and generally shows the strongest alpha oscillatory activity. The first and the last second of each eyes-closed block was discarded to exclude motor activity related to opening and closing the eyes and auditory activity due to the prompt from the speakers. The remaining data was concatenated, resulting in a total of 190s of continuous EEG data. This data was again segmented into 2s epochs, and each epoch containing large amplitude artifacts (>+90 μV, < –90 μV) was excluded from further processing. In 37subjects, more than 50% of trials exceeded this threshold; thus, these subjects were not included in subsequent analyses (see Appendix 2—figure 1). For the remaining data, on average, 2.95% of trials were excluded by this criterion. Power spectral densities (PSDs) were then calculated using Welch’s Method Welch, 1967 implemented in the EEGLab toolbox (Delorme and Makeig, 2004). Zero padding was applied to provide a frequency resolution of 0.25Hz in the 2s sliding time windows within Welch’s algorithm. Averaging the individual PSDs of each window resulted in a smoothed power spectrum that complies with the requirements of the specparam algorithm (Donoghue et al., 2020b) used subsequently (see Specparam algorithm and aperiodic-adjusted alpha power). Additionally, PSDs were transformed to log scale to scale results equal to outputs from the specparam algorithm, which only operates in log power space. In the following, we describe the two approaches to extracting total alpha power and aperiodic-adjusted alpha power together with the aperiodic signal. See Table 7 for an overview of all extracted parameters.

**Table 7.** Overview of extracted parameters.

Parameter	Description
Individual alpha frequency (IAF)	Frequency at maximum power in search window 7–14 Hz
Total canonical alpha power	Averaged log power in the fixed-frequency window [8Hz–13Hz], extracted from the total power spectrum
Total individualized alpha power	Averaged log power in window [- 4 Hz to +2 Hz] relative to IAF, extracted from the total power spectrum
Relative canonical alpha power	Averaged power in the fixed-frequency window [8Hz–13Hz], divided by the average power of the full power spectrum (2–40 Hz), extracted from the total power spectrum
Relative individualized alpha power	Averaged power in window [- 4 Hz to +2 Hz] relative to IAF, divided by the average power of the full power spectrum (2–40 Hz), extracted from the total power spectrum
Aperiodic-adjusted canonical alpha power	Canonical alpha power, extracted from the aperiodic-adjusted log power spectrum
Aperiodic-adjusted individual alpha power	Individualized alpha power, extracted from the aperiodic-adjusted log power spectrum
Aperiodic intercept	Intercept parameter of the aperiodic signal extracted by specparam
Aperiodic exponent	Exponent parameter (i.e. negative slope) of the aperiodic signal extracted by specparam

Note: The term total power spectrum refers to the power spectrum as extracted from the data using Welch’s algorithm. The aperiodic-adjusted power spectrum results from a subtraction of the aperiodic signal from the total power spectrum.

#### Computation of individual alpha peak frequency

The IAF was found by determining the frequency of maximum power between a lower and upper frequency limit. Following previous work, these frequencies limits were set to 7 and 14Hz (Posthuma et al., 2001; Smit et al., 2006). If the peak was located at the border of the search range, no alpha peak was extracted for that subject, and the corresponding data was excluded from further analysis (67subjects, see Appendix 2—figure 1).

#### Extraction of total and relative alpha power

To replicate the results of previous published findings, this standard analysis approach included no adjustment for the aperiodic background signal. If an alpha peak was identified (see Computation of individual alpha peak frequency), individualized total alpha power was extracted by averaging log power in the defined window [–4Hz to+2Hz] relative to the IAF (Klimesch, 1999). This individualized alpha power measure was chosen over a canonically defined alpha range, 8–13Hz (Babiloni et al., 2020), as the shift of the IAF during maturation might introduce a bias when power is averaged within a fixed-frequency window. Canonical alpha band power was also extracted for supplementary analysis.

Relative individualized and relative canonical alpha power were calculated by dividing the corresponding total alpha power values by the average power of the full spectrum (2–40 Hz).

#### Specparam algorithm and aperiodic-adjusted alpha power

**Figure 4.**
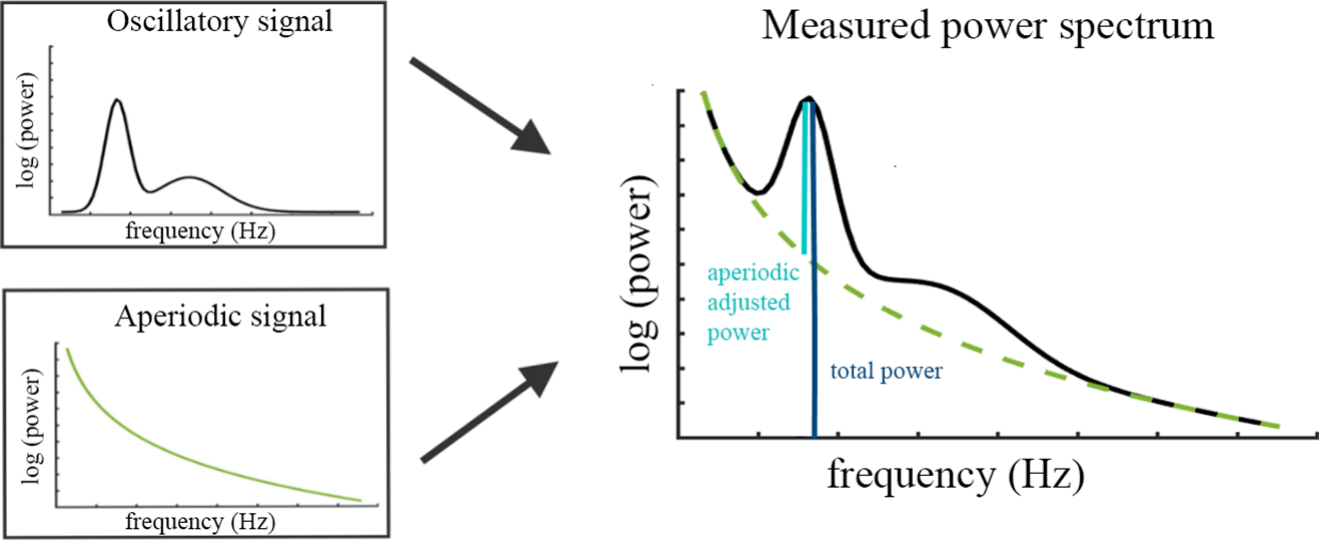
Illustration of the two components (left) superimposed in the measured neural power spectrum (right). The dark blue bar (right) indicates how total power is assessed relative to the absolute zero. The light blue bar represents aperiodic-adjusted power, which is assessed relative to the aperiodic signal.

The specparam algorithm (Donoghue et al., 2020b) parameterizes the neural power spectrum to separate neural oscillations from the aperiodic background signal. The algorithm estimates oscillatory peaks that are superimposed on the aperiodic background signal (see Figure 4) and are therefore measured relative to this rather than to the absolute zero. Consequently, the specparam algorithm parametrizes the PSD by iteratively fitting the aperiodic background curve (L) to the observed smoothed spectral signal, resulting in two parameters: the aperiodic intercept b and the aperiodic exponent χ (i.e. slope, the smaller χ, the flatter the spectrum).

(1) $$L=b-\log\left(k+F^{\chi }\right)$$

In equation 1, F represents the vector of input frequencies and k the knee parameter, which is not further discussed here, as it is set to 0 in the proposed analysis: no bend of the aperiodic component is additionally modeled in the data, which is the default state of the specparam algorithm.

To extract oscillatory components, this aperiodic signal is subtracted from the power spectrum. Gaussians are fitted iteratively to the remaining signal and subsequently subtracted whenever data points exceed two standard deviations of the data. The Gaussians represent the true oscillatory components in the data; if data points are below the specified threshold, they are considered as noise. This results in a data-driven number of Gaussians, each parameterized by the frequency center, power relative to the aperiodic signal and the frequency bandwidth. The power spectrum is therefore modeled as defined by equation 2,

(2) $$P=\;L+\sum_{n=0}^{N}G_{n}+\varepsilon$$

where *G*_n_ represents the *n*^th^ Gaussian and ε the noise not captured by the model. Note that this description of the algorithm is simplified; for a more detailed definition, see Donoghue et al., 2020b.

In the current study, the frequency range of 2–40 Hz of the power spectrum was passed to the algorithm because very low frequencies may lead to overfitting of noise as small bandwidth peaks. The current release (1.0.0) of the specparam toolbox from the github repository (https://github.com/fooof-tools/fooof; Donoghue, 2022) was used. The algorithm was used with these settings: peak width limits: [0.5, 12]; max number of peaks: infinite; minimum peak height: 0; peak threshold: 2 sd above mean; and aperiodic mode: fixed. The resulting periodic signal P (“Oscillatory Signal” in Figure 4) was used to extract aperiodic-adjusted canonical alpha power (average across 8–13 Hz) and aperiodic-adjusted individualized alpha power (average across a [–4 Hz to +2 Hz] window centered on the IAF).

Data was only used for further analysis when the model fit of the specParam model was above a threshold of R^2^ >0.90.

In the analysis of the main HBN sample, an overall high specParam model fit was observed across the sample (mean R^2^=0.9943, sd = 0.0098). Similar model fits were observed in the validation dataset (mean R^2^=0.9941, sd = 0.0120). Model fit was assessed for each of the five occipital electrodes separately (see 4.2.7). Across all subjects, only 11 out of 9,705 model fits were below the cut-off of R^2^ <0.90 in the HBN sample, and 6 out of 1,675 in the validation sample. Consequently, in 99.94% of subjects, all five electrodes could be used to estimate the average occipital periodic and aperiodic parameters. For those subjects with insufficient model fits for specific occipital electrodes, the average occipital periodic and aperiodic parameters were calculated from the average of the remaining electrodes with adequate model fit. In the HBN sample, the numbers of occipital electrodes available in these subjects were: Two (N=1 subject), three (N=1 subject), and four (N=6 subjects). In the validation sample, these numbers of available electrodes were similar: Three (N=2 subject), four (N=2 subjects). No subject was excluded based on the specParam model fit.

A series of control analyses were conducted: The first control analyses indicated a small but significant relation between age and gender with the specParam model fit (see Appendix 4). Controlling for this in the statistical model by adding the specParam model fit as an additional predictor did not change any of the main results (see Appendix 4—table 1). Additional control analyses were subsequently performed to investigate whether possible overfitting of the specParam models (mean R^2^=0.9943, see above) confounded the results. Following the guidelines of Ostlund et al.’s (2022), specParam model fitting parameters and data exclusion criteria were adapted to minimize both overfitting and underfitting of the model (for the details about this approach see Appendix 5). Results were highly consistent with the main results reported in Table 1 and did not indicate any changes to the main results (see Appendix 5—table 1). Finally, a control analysis using the periodic alpha peak power from the specParam algorithm instead of average individualized band power showed highly consistent results and did not change any conclusions (see Appendix 6, Appendix 6—table 1).

#### Electrode cluster analysis

To test the hypothesis derived from literature review, an electrode-cluster-based analysis was performed. This cluster was based on data from the parietal and occipital electrodes, here referred to as the parieto-occipital cluster (see Figure 1). These electrodes were chosen because of the strong prominence of Oz and Pz electrodes in research on EEG alpha oscillations (Klimesch, 1999) and previous findings for age effects on alpha band power in these electrodes (Clarke et al., 2001; Cragg et al., 2011; Gasser et al., 1988). To account for individual anatomical differences, the following electrodes were selected to create a more robust cluster: E72 (POz), E75 (Oz), E62 (Pz), E67 (PO3), E77 (PO4). All parameters described above were extracted for each electrode and subsequently averaged within this cluster. Prior to statistical analyses, data was excluded from further processing if any extracted parameter exceeded a threshold of three standard deviations above or below the mean of the sample (excluded N=30, mean age = 12.58, sd = 3.92). See Appendix 2—figure 1 for a detailed flow chart of all exclusion criteria and the resulting sample size.

#### Statistical analysis

Bayesian generalized linear mixed models were formulated using the brms R package (Bürkner, 2017). Statistical models were separately fitted to the full sample and the subsampled dataset of subjects without any given diagnosis. For both samples, the predictor variable was age, and the covariates gender, EHI, and site were added. In the full sample, a categorical variable for diagnosis was added (no diagnosis, ADHD diagnosis, other diagnosis) to account for the high prevalence of ADHD in this dataset (N_no diagnosis_ = 190, N_ADHD diagnosis_ = 1038, N_other diagnosis_ = 542). However, the focus of this paper is on brain maturation; thus, this information is only included to control for possible confounding effects of psychiatric disorders.

To determine the best model for each analysis, multiple models were fitted with varying degrees of interaction terms and compared using the Watanabe Akaike Information Criteria (WAIC, Wantanabe, 2013). Additionally, expected log pointwise predictive density (ELDP, i.e. the expected predictive accuracy of the model) was calculated using the R function ‘loo_cmpare’ (Vehtari et al., 2017), yielding consistent results. For an overview of all models tested and the resulting WAIC and ELDP, see Supplementary file 1.

The final models were these:

For the subsample of subjects without any given diagnosis:

(3) $$\left[dv^{\prime }s\right]∼age+gender+EHI+site$$

For the full sample:

(4) $$\left[dv^{\prime }s\right]∼age+gender+diagnosis+EHI+site$$

The multivariate models defined above were fitted each to a set of dependent variables: Total individualized alpha power, aperiodic-adjusted individualized alpha power, relative individualized alpha power, aperiodic intercept, aperiodic slope and IAF. Additionally, for supplementary analyses, model two was also fitted to measures of canonical alpha power (i.e. total canonical alpha power, relative canonical alpha power and aperiodic-adjusted canonical alpha power).

To correct for multiple comparisons, the significance level was adjusted. We assumed a high correlation between the total of 9 outcome variables (including the canonical alpha power measures, see Supplementary file 2C), as many of the dependent variables represent various characteristics of the individual alpha oscillations. To account for this, we first calculated the effective number of tests of all dependent variables using Nyholt’s approach (Nyholt, 2004). Following this approach, the significance level (0.05) was then adjusted using the Šidák correction (Nyholt, 2004). Subsequently, the credible intervals (CIs) of the posterior distributions were calculated from the newly estimated levels of significance. The resulting significance level was 0.0083, yielding 99.17% credible intervals. We refrained from calculating Bayes factors for point estimates as evidence of the effect being zero or unequal to zero, as these Bayes factors, which are based on the Savage–Dickey ratio, depend strongly on the arbitrary choice of the prior distribution of each effect. Instead, we considered a model parameter significant if its 99.17% CI did not include zero. In line with Gelman’s recommendations (Gelman et al., 2007), predictors and outcome variables of the Bayesian regression model were scaled as follows: Each numeric parameter (age, EHI, IAF, total canonical alpha power, total individualized alpha power, relative canonical alpha power, relative individualized alpha power, aperiodic-adjusted canonical alpha power, aperiodic-adjusted individualized alpha power, aperiodic intercept, and aperiodic slope) was scaled to provide a mean of 0 and standard deviation 0.5. Uninformative Cauchy priors were used (mean = 0, sd = 2.5), as proposed by Gelman (Gelman et al., 2007).

### Anatomical thalamic measures

Total and aperiodic-adjusted alpha power measures were further related to the thalamic volume and the fractional anisotropy (FA) of the thalamic radiation.

#### MRI data acquisition

DTI and T1-weighted scans were acquired at three acquisition sites: Staten Island (SI), Rutgers University Brain Imaging Center (RUBIC), and CitiGroup Cornell Brain Imaging Center (CBIC). For the full scanning protocol and site-specific scanning parameters see Alexander et al., 2017 and Appendix 3A.

#### DTI preprocessing

DTI data was processed using the FMRIB Software Library (FSL) version 6.0.4 (Jenkinson et al., 2012) following the ‘Diffusion parameter EStImation with Gibbs and NoisE Removal’ (DESIGNER) pipeline (Veraart et al., 2018). Detailed descriptions of all processing steps are available in Appendix 3B and the entire preprocessing code is available at https://github.com/sdziem/DTIPreprocesingPipeline (copy archived at swh:1:rev:e4b48053d150976e1036a46b25870d7973965efc; Dziemian, 2021).

In brief, DTI data preprocessing included denoising (Veraart et al., 2016) followed by correction for Gibbs artefacts (Kellner et al., 2016). Preprocessing continued with brain extraction with an FA threshold of 0.1 (Smith, 2002) and state-of-the-art correction for eddy current-induced distortions in-scanner head motion (Andersson et al., 2017; Andersson et al., 2016; Andersson and Sotiropoulos, 2016). Next, outlier detection of MRI parameters and robust parameter estimation (Collier et al., 2015), tensor fitting, and extraction of diffusivity measures using weighted linear least squares estimation was applied (Fieremans et al., 2011; Veraart et al., 2011; Veraart et al., 2013).

Using automating fiber-tract quantification (AFQ, v.1.1) (Yeatman et al., 2012), which implements a deterministic streamline tracking algorithm (Basser et al., 2000; Mori et al., 1999), we extracted objective and reliable FA values along the left and right thalamic radiation reflecting the tracts’ white matter integrity (Wassermann et al., 2011; Yeatman et al., 2011; Yeatman et al., 2012). In brief a co-registered T1-weighted scan was used to define anatomical regions of interest as seeds for consecutive tractography. Whole-brain tractography was computed using the following settings: FA threshold of 0.2, an FA mask threshold of 0.3, angle threshold of 35°. Next, the left and right thalamic radiations were segmented by distinct anatomical regions of interest as defined in a WM atlas (Wakana et al., 2007) in the co-registered T1-weighted scan. Fiber tract probability maps (Hua et al., 2008) were used to refine the tracts according to the likelihood of a fiber belonging to the tract. Fiber tract cleaning and outlier removal was performed using four standard deviations from the mean tract length and five standard deviations in distance from the tract core as removal criteria (Yeatman et al., 2012).

Each fiber belonging to the left and right thalamic radiation was sampled at 100 equidistant nodes (Yeatman et al., 2012). FA values were calculated for each segment along the tract as the sum of FA values of corresponding fibers weighted by the probability of the given fiber being part of the tract (Yeatman et al., 2012). This yields FA tract profiles for 100 equidistant segments for each participant’s left and right thalamic radiation. From these profiles, we calculated the tract-wise mean FA, which provides a more reliable quantification of the tract’s white matter integrity (Carlson et al., 2014; Luque Laguna et al., 2020).

Additionally, to ensure high imaging quality, each participant’s whole brain tractography underwent visual inspection for incomplete tractographies, gross artefacts, and misalignments of scans. Visual inspection was performed by a rater blind to the demographics of the participants. Only data rated as ‘good’ was included in the final statistical analysis.

#### T1-weighted preprocessing

T1-weighted scans were preprocessed in parallel (Tange, 2011) with FreeSurfer (version 6.0.0) (http://surfer.nmr.mgh.harvard.edu/). Subcortical volumetric segmentation of the left and right thalamus was computed using the function ‘recon-all’. Details on the subcortical volumetric segmentation procedure have been reported previously (Fischl et al., 2002; Fischl et al., 2004; Han et al., 2006) and are provided in Appendix 3C. Briefly, processing included (1) correction for intensity non-uniformity, (2) Talairach transformation, (3) intensity normalization, (4) skull stripping, and (5) automated subcortical segmentation and labeling based on the default Gaussian Classifier Atlas (GCA) (Fischl et al., 2002; Fischl et al., 2004; Han et al., 2006). The validity of automated segmentation of the thalamus has been verified previously (Keller et al., 2012). Bilateral thalamic volumes (i.e. Left-Thalamus-Proper, Right-Thalamus-Proper) and total intracranial volume (i.e., EstimatedTotalIntraCranialVol) were extracted for subsequent analysis.

#### Relation to alpha power

To investigate the relation between thalamic volume and the FA of the thalamic radiation, Bayesian regression models similar to those described in Statistical analysis were fitted, adding the thalamic volume and left and right thalamic radiation predictors while controlling for total intracranial volume, age and gender:

(5) $$\left[dv^{\prime }s\right]∼thalamic\;volume+total\;intracranial\;volume+\;age+gender$$

(6) $$\left[dv^{\prime }s\right]∼left\;thalamic\;radiation+total\;intracranial\;volume+\;age+gender$$

(7) $$\left[dv^{\prime }s\right]∼right\;thalamic\;radiation+total\;intracranial\;volume+\;age+gender$$

Due to collinearity issues, separate models were fitted for the left and right thalamic radiation. The dependent variables for Models 4, 5, and 6 were total individualized alpha power, aperiodic-adjusted individualized alpha power, relative individualized alpha power and the aperiodic intercept and slope. As described above, the numeric predictors and outcome variables were scaled to provide a mean of 0 and standard deviation 0.5. Uninformative Cauchy priors were used (mean = 0, sd = 2.5), as proposed by Gelman (Gelman et al., 2007). The models were fitted to the subset of the full sample, for which DTI for thalamic radiation and structural MRI for thalamic volume and total intracranial volume was available. This yielded a sample size of 851 subjects (mean age = 11.19, sd = 3.58, age range = 5.04–21.89, 294 female). To correct for multiplicity, the significance level was adjusted as described in section Statistical analysis. For the five outcome variables, the resulting significance level was 0.0148, yielding 98.52% credible intervals.

### Relation of alpha power to Flanker task scores

Post hoc analyses were performed to investigate the relationship between the different measures of alpha power and attentional performance. The Flanker task of the National Institutes of Health Toolbox Cognition Battery (Gershon et al., 2013) was employed as a measure of attentional performance. In each of the 40 trials of this task, a set of stimuli is presented to participants, who are asked to indicate the direction (left or right) of the central stimulus. These stimuli are either arrows, for participants of the age 8 years or older, or fish, for children younger than 8 years. Therefore, participants need to focus attention on the central stimulus and suppress information from surrounding stimuli, which may be congruent or incongruent to the direction with the central stimulus. Thus, the task aims to measure inhibitory control and visual selective attention. This data was collected as part of the HBN study. Participants performed a computerized version of the task in a separate assessment without neurophysiological recording. The score was calculated based on a combined measure of accuracy across trials and reaction time (Zelazo et al., 2014). Age standardized scores were automatically extracted by the test software (for more details, see National Institutes of Health and Northwestern University, 2022). The final sample size, for which both task data and EEG data were available, was N=1,757 (age range, 5–22 years, mean age = 10.81, sd = 3.44).

The score in the Flanker task was used as the predictor in the linear models. The univariate linear models controlled for age, gender, and handedness (EHI) and were defined as:

alpha power∼Flanker score+ age+gender+EHI

All continuous predictors and outcome variables were standardized (z-transformed). The models were fitted separately for three outcome variables: total individualized alpha power, relative individualized alpha power, and aperiodic-adjusted individualized alpha power. To account for multiple comparisons with three different measures of alpha power, the effective number of tests was calculated using Nyholt’s approach, and Šidák correction was applied to adjust the significance level of 0.05 (Nyholt, 2004), yielding a corrected significance level of 0.0264.

### Validation study

To validate the results from the analysis of the main dataset, the same analyses (sections Experimental setup and procedure - Statistical analysis) were applied to the second dataset of 369 subjects. Before the analysis pipeline was performed on this dataset, all analyses were preregistered in https://osf.io/7uwy2. In this dataset, eyes-closed resting-state EEG (mean length = 260 s, sd = 28.4 s) was recorded at a sampling rate of 500 Hz using a NeuroAmp ×23 with a PC-controlled 19-channel electroencephalographic system. Electrodes were placed according to the international 10–20 system using an electrode cap with tin electrodes (Electro-cap International Inc, Eaton, Ohio, USA). Data was referenced to the linked earlobes, and impedances were kept below 5 kΩ. Data was filtered between 0.5 and 50 Hz, and the same artifact correction as described in Electroencephalography recording and preprocessing was subsequently applied. The electrodes used for the parieto-occipital electrode cluster were Pz, P3, P4, O1, and O2. The same exclusion criteria as described above were applied. In this dataset, 34 subjects were excluded due to bad EEG data quality. Additionally, 11 subjects were excluded due to missing demographic data, 10 subjects had no detectable IAF, and 5 subjects were excluded due to outlier detection. This yielded a final sample size of 310 subjects (see Table 6 for an overview of the characteristics of the final sample). The parameters were scaled as described in Statistical analysis, and the same uninformative Cauchy priors were used. An additional analysis combined evidence across the two datasets by extracting the posterior distributions for age and gender effects from the analyses of the HBN datasets and approximating them to the best-fitting distribution using the fitdistrplus R package (Delignette-Muller and Dutang, 2015). Subsequently, the statistical models were refitted using these extracted posteriors as priors for the analyses of the validation dataset.

## Figures and Tables

**Appendix 1—figure 1.**
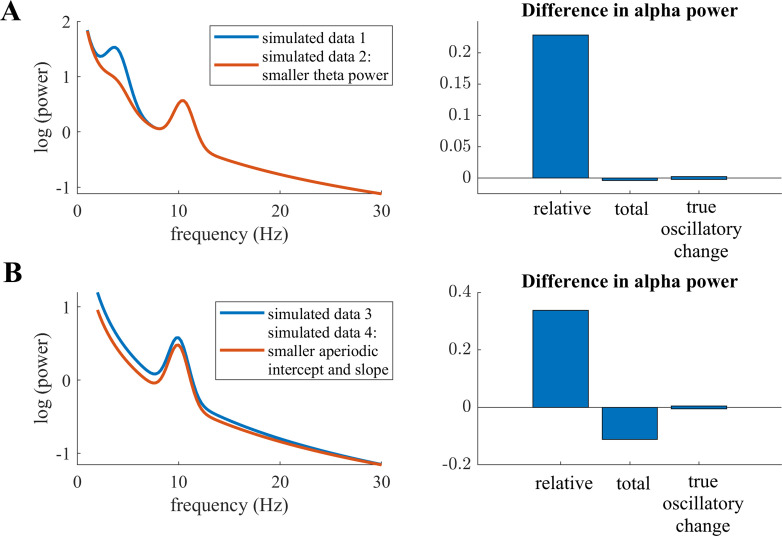
Visualizations of possible fallacies in total and relative power measures in simulated data. Bar plots on the right indicate the difference in alpha power between simulated data 2 and simulated data 1 in A, and simulated data 4 to simulated data 3 in B. (**A**) Two simulated power spectra with identical true alpha oscillatory power. The high amplitude oscillation in the theta range (~5 Hz) in simulated data 1 conflates results in relative power differences in the alpha band. (**B**) Two simulated power spectra with identical alpha oscillatory power. Here, differences in the aperiodic intercept and slope between the two signals conflate results in total and relative power differences in the alpha band.

**Appendix 2—figure 1.**
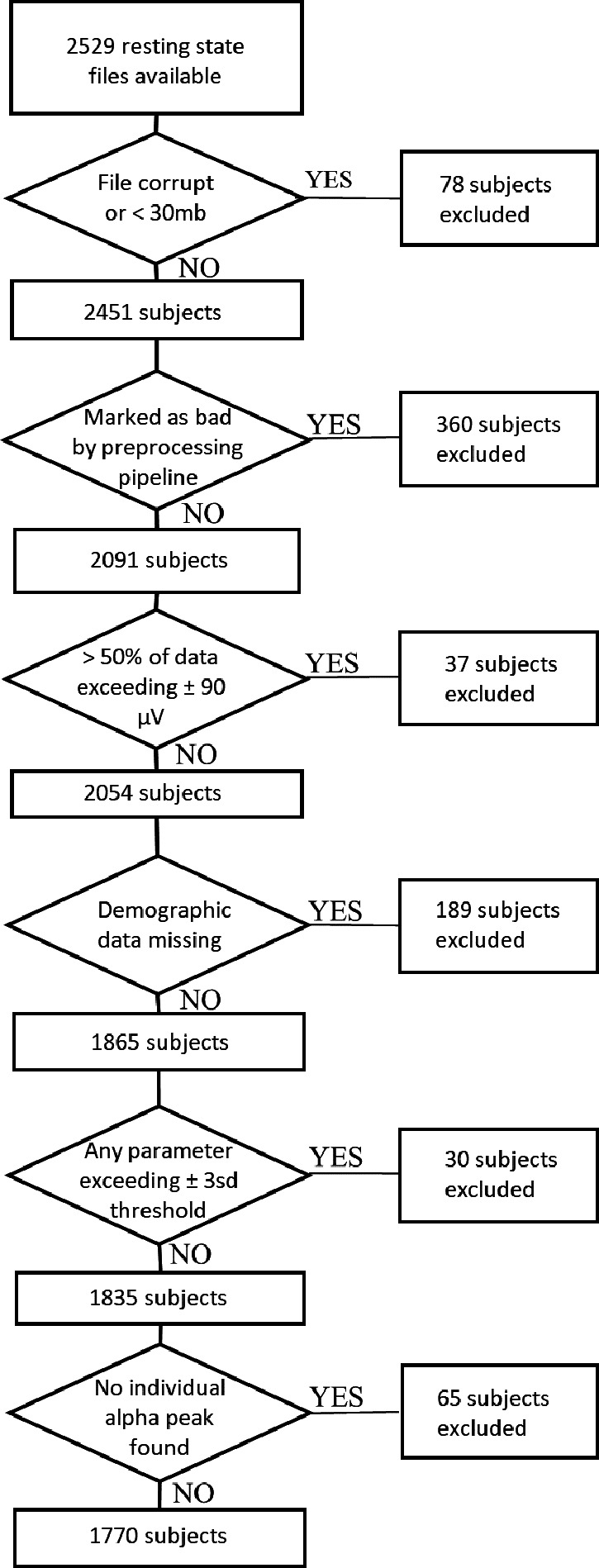
Flow chart of exclusion criteria applied to the main HBN dataset, yielding 1770 subjects plotted in Figure 1 and used for the statistical analyses.

## Data Availability

All data generated or analyzed during this study are included in the manuscript and supporting file; Source Data files have been provided for all figures. All data analysis code is additionally provided in: https://osf.io/4nzyk/. This repository further contains all extracted EEG features and demographics which were used in the statistical models. The raw data for the HBN sample (N = 2529) is available here: (http://fcon_1000.projects.nitrc.org/indi/cmi_healthy_brain_network/sharing_neuro.html). The raw data from the validation data set (N = 369) presented in this article are not readily available for the public research community, because we do not have permission from the participants to share the raw data. We can only share derivatives of the validation data. Requests to access the raw validation datasets should be directed to NL, n.langer@psychologie.uzh.ch. The following previously published dataset was used: AlexanderL 2017Neuroimaging Data Accessfconcmi_healthy_brain_network

## Additional files

Supplementary file 1. Supplementary table presenting model comparison results for the main statistical analysis of the HBN sample.

Supplementary file 2. Supplementary statistics tables of the main dataset. A: Bayesian regression model results of HBN subsample of subjects without any given diagnosis.B: Bayesian regression model results of full HBN sample with categorical sub-diagnosis predictor.C: Bayesian regression model results for canonical alpha power measures in the full HBN sample.

Supplementary file 3. Supplementary statistics tables of the validation dataset. A: Validation study: Bayesian regression model results of subjects without any given diagnosis, using uninformative priors.B: Validation study: Bayesian regression model results using informative priors extracted from the main HBN analysis.

Supplementary file 4. Supplementary analysis results on global white matter integrity extracted from DTI. Bayesian regression model investigating the influence of global white matter integrity on aperiodic signal parameters in the HBN dataset.

Transparent reporting form

## Appendix 1

### Simulating confounds in total and relative alpha power

To highlight confounding factors in the analysis of age-related changes in relative alpha power, simulations were performed. Therefore, power spectra were simulated as defined in the specParam algorithm (see equation 1 and 2, section 2.2.6). Gaussians representing neural oscillations were further defined as in Donoghue et al., 2020b:

$$G_{n}=a\;\times \exp\left(\frac{-\;{\left(F-c\right)}^{2}}{2w^{2}}\right)$$

First, two power spectra were generated sharing the identical aperiodic signal component (intercept = 1.82, exponent = 1.99). The same alpha oscillation was added to both power spectra, being defined by the center frequency c=10.5 Hz, the power a = log_10_ (6), and the bandwidth w=1. A larger theta oscillation as added to the first power spectrum (simulated data 1, c=4, a = log_10_ (7.5) and w=1.1) than to the second power spectrum (simulated data 2, c=4, a = log_10_ (2) and w=1.1). Resulting power spectra are plotted in Appendix 1—figure 1A.

For the second simulated case, two power spectra were generated, which differed in the aperiodic signal component (simulated data 3: intercept = 1.8, exponent = 2.0; simulated data 4: intercept = 1.5, exponent = 1.8). The same alpha oscillation was added to both simulated power spectra (c=10, a = log_10_ (6) and w=1). Resulting power spectra are visualized in Appendix 1—figure 1B. For both scenarios, differences in relative and total individualized alpha power were calculated.

## Appendix 2

Appendix 2—figure 1 visualizes the detailed exclusion criteria and their effect on the sample size of the main HBN dataset.

## Appendix 3

### A: Site-specific scanning parameters

For detailed scanning protocols for all three acquisition sites see here and Alexander et al., 2017.

#### Staten island (SI) scanning site

Scanning at site SI was performed in a mobile trailer with a 1.5T Siemens Avanto system and a Siemens 32-channel head coil, 32 RF receive channels and 45 mT/m gradients. The University of Minnesota Center for Magnetic Resonance Research (CMRR) simultaneous multi-slice echo planar imaging sequence was used. DTI imaging was acquired at 72 slices, with a resolution of 2 × 2 x 2 mm in 64 diffusion directions and b-values of 0, 1,000 and 2000 s/mm^2^. Further DTI parameters were TR = 3110ms, TE = 76.2ms, flip angle of 90 degrees and threefold multiband acceleration.

T1-weighted imaging was acquired at 176 slices with a resolution of 1 × 1 x 1 mm and scanning parameters of TR = 2730ms, TE = 1.64ms, TI = 1000ms and a flip angle of 7 degrees.

#### Rutgers University Brain Imaging Center (RUBIC) scanning site

At the RUBIC site, scans were acquired with a Siemens 3T Tim Trio MRI scanner, a Siemens 32-channel head coil using the CMRR simultaneous multi-slice echo planar imaging sequence. DTI imaging comprised 72 slices with a resolution of 1.8 × 1.8 x 1.8 mm. Further DTI specifications were: TR = 3320ms, TE = 100.2ms, a flip angle of 90 degrees, threefold multiband acceleration, 64 diffusion directions and b-values of 0, 1,000 and 2000 s/mm^2^.

For T1-weighted imaging 224 slices at a resolution of 0.8 × 0.8 x 0.8 mm were acquired with TR = 2500ms, TE = 3.15ms, TI = 1060ms and flip angle of 8 degrees.

#### CitiGroup Cornell Brain Imaging Center (CBIC) scanning sites

The CBIC site was equipped with a 3T Prisma scanner and a Siemens 32-channel head coil using the CMRR simultaneous multi-slice echo planar imaging sequence. DTI was acquired at 81 slices with a resolution of 1.8 × 1.8 x 1.8 mm in 64 diffusion directions and b-values of 0, 1000, and 2000 s/mm^2^. Further scanning parameters were set to TR = 3320ms, TE = 100.2ms, a flip angle of 90 deg, threefold multiband acceleration. The parameters for T1-weighted imaging at CBIC were identical to the RUBIC site.

### B: DTI Preprocessing

The DTI preprocessing pipeline included the following steps: (1) Denoising was performed using the ‘MPdenoising’ function employing a four-dimensional image denoising and noise map estimation algorithm (Veraart et al., 2016). (2) Gibbs artefacts were removed from the denoised images using the ‘unring’ function with the default parameters (Kellner et al., 2016). The following steps were executed using the FMRIB Software Library (FSL) version 6.0.4 (Jenkinson et al., 2012). (3) We used the FSL Brain Extraction Tool (BET) to obtain a binary brain mask from the whole head image using a fractional anisotropy threshold of 0.1 (Smith, 2002). (4) Eddy current-induced distortions and in-scanner head motion artefacts were removed using the FSL tool ‘eddy_cuda’ (Andersson et al., 2016; Andersson et al., 2017; Andersson and Sotiropoulos, 2016). For this tool the settings were as follows: 8 iterations, smoothing full-width-half-max parameters of [10, 6, 4, 2, 0, 0, 0, 0] for each respective iteration, enabled outlier detection and replacement for slice-wise and multiband group outliers and additional 8 iterations for slice-to-volume correction. (5) Outlier detection of MRI parameters and robust parameter estimation was performed with the function ‘irlls’ applying iterative reweighted linear least squares (Collier et al., 2015). (6) Using the function ‘dki_fit’ (Veraart et al., 2013) we performed tensor fitting and extraction of diffusivity measures based on weighted linear least squares estimation. (7) For the extraction of DTI parameters we used the function ‘dki_parameters’ (Veraart et al., 2011). (8) To calculate white matter tract integrity metrics we applied the function ‘wmti_parameters’ (Fieremans et al., 2011). (8) AC-PC aligned nifti image was created using the function ‘mrAnatAutoAlignAcpcNifti’ (https://github.com/vistalab/vistasoft, Vistalab; Cardinal, 2022, Stanford University, Stanford, CA). The output of this preprocessing pipeline was the input for tractography using Automating Fiber-Tract Quantification (AFQ, v0.1) (Yeatman et al., 2012). (9) The parameters for the function ‘AFQ_Create’ were: ‘cutoff’ of [5 95], FA threshold of 0.2, FA mask threshold of 0.3, and angle threshold of 35 degrees. (10) We used the ‘AFQ_run’ function to calculate individual white matter tract profiles of FA. (11) Based on these tract profiles, we calculated a mean FA value which is the average FA of 100 equidistant nodes along clipped predefined regions of the left and the right thalamic radiation, respectively.

### C: T1-weighted preprocessing

T1-weighted scans were preprocessed with FreeSurfer (version 6.0.0) (http://surfer.nmr.mgh.harvard.edu/). Subcortical volumetric segmentation of the left and right thalamus was computed using the function recon-all (Fischl et al., 2002; Fischl et al., 2004; Han et al., 2006). First, the T1-weighted scan was corrected for intensity non-uniformity with Non-parametric Non-uniform intensity Normalization. Second, the Talairach transformation was computed which is an affine transform to the MNI305 atlas. Third, intensity normalization was applied, which corrects deviations in intensity for later intensity-based segmentation. All voxel’s intensity was scaled such that the white matter mean intensity was 110. Fourth, skull tissue was removed from the intensity normalized image and a brain mask was created. Next automatic subcortical segmentation was performed. For this, the intensity non-uniformity corrected (NU-corrected) image was co-registered to the Gaussian Classifier Atlas (GCA). Based on the GCA model the image was first normalized and nonlinearly transformed to the GCA atlas. In a next step, regions corresponding to the neck were removed from the NU-corrected image. Further, a transformation was applied of the NU-corrected image without the neck to the GCA image containing the skull. Lastly, subcortical structures were labeled in accordance with the GCA model and statistics on the segmented subcortical structures computed and summarized.

## Appendix 4

### Control analysis: Influence of specParam model fit

Control analyses were performed to investigate the relationship between the specParam model fit and age, gender and diagnoses (no diagnoses, ADHD, other diagnosis) controlling for recording site and handedness (EHI) in the full HBN dataset. A linear model was formulated as

specParam model fit∼age+gender+diagnosis+EHI+site

No significant effects were found in diagnosis, EHI or site; however, age (*b*=–0.0002, p=6.63e- 10) and gender (*b* female = –0.0015, p=7.73e-15) showed significant negative associations with the model fit.

To rule out possible confounding effects of the specParam model fit in the analysis of alpha power and aperiodic signal components, additional control analyses were performed, adding a predictor labeled ‘specParam model fit’ to the main statistical analysis (equation 2, section 2.2.8). Therefore, for the main HBN dataset, a multivariate Bayesian regression model (brms) was defined as

$$\left[dv^{\prime }s\right]\;∼\;age*gender+diagnosis+EHI+site+specParam\;model\;fit$$

As summarized in Appendix 4—table 1 and a significant association of the specParam model fit with the magnitude of the alpha power measures and the aperiodic signal parameters was observed in the main HBN dataset. However, the original results and conclusions remained unaffected: A significant negative effect of age was found on the aperiodic intercept and slope and on total individualized alpha power. Aperiodic-adjusted and relative individualized alpha power and the alpha peak frequency showed a positive significant association with age. All these six dependent variables showed a significant association with gender (i.e. smaller values for females as compared to males) and no significant associations with the clinical diagnosis.

**Appendix 4—table 1.** Main HBN dataset: Bayesian regression model controlling for possible influence of the specParam model fit on effects of age, gender, and diagnosis on the outcome variables.

Outcome	β_predictor_ [CI]					
	Age	Gender	Diagnosis: ADHD	Diagnosis: Other	Age*gender	specParam model fit
Alpha peak frequency	0.41 [0.34 0.49]	–0.08 [–0.14 –0.02]	–0.07 [–0.17 0.02]	–0.06 [–0.16 0.04]	–0.04 [–0.16 0.08]	–0.04 [–0.10 0.02]
Total individualized alpha power	–0.30 [–0.37 –0.23]	–0.34 [–0.37 –0.23]	0.02 [–0.08 0.11]	0.01 [–0.09 0.12]	0.15 [0.04 0.27]	0.13 [0.07 0.19]
Relative individualized alpha power	0.18 [0.10 0.25]	–0.31 [–0.38 –0.25]	0.00 [–0.10 0.10]	0.00 [–0.10 0.11]	–0.03 [–0.15 0.09]	0.17 [ 0.11 0.23]
Aperiodic-adjusted individualized alpha power	0.27 [0.20 0.34]	–0.35 [–0.41 –0.29]	–0.03 [–0.12 0.07]	–0.02 [–0.13 0.08]	–0.04 [–0.16 0.08]	0.15 [ 0.09 0.20]
Aperiodic intercept	–0.54 [–0.60 –0.48]	–0.31 [–0.36 –0.26]	0.00 [–0.08 0.09]	–0.01 [–0.09 0.08]	0.11 [0.02 0.21]	0.23 [0.18 0.27]
Aperiodic slope	–0.43 [–0.49 –0.37]	–0.31 [–0.36 –0.26]	–0.02 [–0.10 0.06]	–0.03 [–0.11 0.06]	–0.01 [–0.10 0.09]	0.32 [ 0.27 0.37]

Note: CI = 98.97% Credible Interval, gender variable is dummy coded: 1=female, 0=male.

## Appendix 5

### Control analysis: SpecParam fitting procedure following guidelines of Ostlund et al., 2022

To rule out potential resulting confounding effects of specParam model overfitting (mean R-squared=0.9943, sd = 0.0098 in the full HBN sample) and the cut-off defined a priori for exclusion of bad specParam model fits (R^2^ <0.9), additional control analyses were performed following the procedure described in the guidelines by Ostlund et al., 2022. Following these guidelines, we randomly subsampled 10% of the HBN dataset. First, we applied the default specParam fitting parameters as previously in the initial submission (peak width limits: [0.5 12], max number of peaks: infinite, minimum peak height: 0, peak threshold: 2 sd above mean, aperiodic mode: fixed). Subsequently, the proportion of model underfits and overfits were determined using the cut-offs mean absolute error (MAE)>0.1 for underfitting and MAE <0.025 for overfitting. The definitions of these MAE cut-offs were adopted from the example analysis in Ostlund et al., 2022, who investigated a sample of children (mean age = 9.97, sd = 0.95). Applying these cut-offs yielded significant loss of data in the subsample analyzed (5.3% overfitting, 0.001% underfitting). Consequently, to minimize overfitting, the model fit parameters were changes as suggested in Ostlund et al., 2022: peak width limits: [1 8]; max number of peaks: 6; minimum peak height: 0.1; peak threshold: 2 sd above mean; aperiodic mode: fixed. Applying specParam again to data of the same 10% subsample of the HBN data yielded very little loss of data (0.001% underfitting, 0.4% overfitting).

Subsequently, all analyses described in 4.2.3–4.2.8 were repeated for the full HBN dataset, applying these updated specParam fitting parameters. With these parameter settings, 0.2% of all fitted specParam models were underfitting, and 0.5% were overfitting in the full sample, applying the new exclusion criteria (MAE >0.1 or MAE <0.025).

The control analysis showed highly consistent results compared to the previous analysis reported in Table 1, see Appendix 5—table 1 for detailed results.

**Appendix 5—table 1.** Bayesian regression model results of the main dataset. Adjusted specParam fitting parameters (peak width limits: [1 8]; max number of peaks: 6; minimum peak height: 0.1; peak threshold: 2 sd above mean; aperiodic mode: fixed) and data exclusion criteria of specParam fit: MAE >0.1 or MAE <0.025.

Outcome	β_predictor_ [CI]				
	Age	Gender	Diagnosis: ADHD	Diagnosis: Other	Age*gender
Alpha peak frequency	0.42 [0.34 0.49]	–0.05 [–0.12 –0.01]	–0.07 [–0.16 0.04]	–0.06 [–0.16 0.05]	–0.01 [–0.15 0.10]
Total individualized alpha power	–0.33 [–0.40 –0.26]	–0.36 [–0.42 –0.30]	0.01 [–0.09 0.10]	0.02 [–0.08 0.12]	0.14 [0.03 0.26]
Relative individualized alpha power	0.15 [0.07 0.22]	–0.34 [–0.40 –0.28]	–0.01 [–0.11 0.09]	0.01 [–0.09 0.11]	–0.04 [–0.16 0.09]
Aperiodic-adjusted individualized alpha power	0.24 [0.17 0.32]	–0.37 [–0.43 –0.31]	–0.03 [–0.13 0.07]	–0.02 [–0.11 0.08]	–0.04 [–0.16 0.08]
Aperiodic intercept	–0.57 [–0.63 –0.518]	–0.35 [–0.40 –0.30]	–0.01 [–0.09 0.07]	–0.01 [–0.10 0.07]	0.09 [–0.01 0.19]
Aperiodic slope	–0.47 [–0.53 –0.40]	–0.37 [–0.42 –0.32]	–0.03 [–0.12 0.05]	–0.04 [–0.13 0.05]	–0.04 [–0.15 0.06]

Note: Credible Interval (CI)=99.17%.

## Appendix 6

### Control analysis: Analysis of specParam periodic alpha peak power

The present results indicated an age-related increase in aperiodic-adjusted alpha power when averaging power from the aperiodic-adjusted power spectrum in a frequency window commonly applied in literature ([–4 Hz 2 Hz] centered around the individual alpha peak frequency, e.g., Klimesch, 1999). Alternatively, as proposed by Donoghue et al., 2020b, the periodic peak in the alpha range could be extracted from the specParam algorithm as an indicator of individual alpha power. A control analysis was performed on the main HBN dataset to investigate whether results are consistent when applying both periodic measures of alpha power. Therefore, the highest Gaussian oscillatory peak detected by the specParam algorithm was found in a search window (7.5 Hz to 13.5 Hz) and its height was extracted as the individual alpha power. The analyses described in 4.2 were repeated, using the specParam oscillatory peak height instead of the average of the aperiodic-adjusted power spectrum in the individual alpha range. Results are highly consistent across the two approaches. The results for the specParam periodic alpha peak power approach are: *b*_age_ = 0.20, CI = [0.12, 0.27], *b_gender_* = –0.30, CI = [-0.37,–0.24]. The results for aperiodic-adjusted individual alpha band power approach found: *b*_age_ = 0.23, CI = [0.16, 0.30], *b_gender_* = –0.39, CI = [-0.45,–0.33]. This is not surprising as the average aperiodic-adjusted power spectrum in the individual alpha range and specParam periodic alpha peak power are highly correlated (*r*=0.92). Detailed results of the control analysis are reported in Appendix 6—table 1.

**Appendix 6—table 1.** Bayesian regression model results of the main HBN dataset, using the specParam periodic alpha peak parameter.

Outcome	β_predictor_ [CI]			
	Age	Gender	ADHD diagnosis	Age*gender
Alpha peak frequency	0.42 [0.35 0.49]	–0.07 [–0.14 –0.02]	–0.07 [–0.17 0.02]	–0.04 [–0.15 0.08]
Total individualized alpha power	–0.31 [–0.39 –0.24]	–0.37 [–0.42 –0.30]	0.02 [–0.08 0.11]	0.14 [0.02 0.25]
Relative individualized alpha power	0.16 [0.08 0.23]	–0.34 [–0.41 –0.28]	–0.01 [–0.11 0.09]	–0.05 [–0.17 0.07]
specParam periodic alpha peak power	0.20 [0.12 0.27]	–0.30 [–0.37 –0.24]	–0.01 [–0.11 0.08]	–0.05 [–0.17 0.07]
Aperiodic intercept	–0.56 [–0.62 –0.50]	–0.35 [–0.41 –0.30]	0.00 [–0.09 0.08]	0.09 [–0.01 0.19]
Aperiodic slope		–0.38 [–0.43 –0.33]	–0.03 [–0.11 0.06]	–0.04 [–0.15 0.06]

Note: CI = 99.17% Credible Interval; gender variable is dummy coded: 1=female, 0=male.
