# Supplementary material for: Decomposing the role of alpha oscillations during brain maturation
Source: eLife. 2022 Aug 25;11:e77571. doi: 10.7554/eLife.77571 (PMC9410707; doi:10.7554/eLife.77571)
Supplement: Supplementary file 1. [file elife-77571-supp1.docx]

**Supplementary table presenting model comparison results for the main statistical analysis of the HBN sample.**

*Model comparison results*

|  | WAIC | ELDP_diff (se) |
| --- | --- | --- |
| Subsample without any given diagnosis |  |  |
| $\left[ dv^{'}s \right]\sim age+gender+EHQ+site$ | -183.03 | 0.00 (0.00) |
| $\left[ dv^{'}s \right]\sim age*gender+EHQ+site$ | -172.19 | -5.42 (4.02) |
| Full Sample, categorical diagnosis variable |  |  |
| $\left[ dv^{'}s \right]\sim age+gender+diagnosis+ EHQ+site$ | -1534.40 | -19.70 (7.98) |
| $\left[ dv^{'}s \right]\sim age*gender+diagnosis+ EHQ+site$ | -1573.79 | 0.00 (0.00) |
| $\left[ dv^{'}s \right]\sim age+gender*diagnosis+ EHQ+site$ | -1526.36 | -23.72 (8.16) |
| $\left[ dv^{'}s \right]\sim age*diagnosis+age* gender+ EHQ+site$ | -1561.44 | -6.18 (2.99) |
| $\left[ dv^{'}s \right]\sim age*gender*diagnosis+ EHQ+site$ | -1559.82 | -6.99 (8.42) |

*Note:* Dependent variables (dv’s) used for the model comparisons are : Total individualized alpha power, aperiodic-adjusted individualized alpha power, relative individualized alpha power, aperiodic intercept, aperiodic slope and IAF. WAIC refers to the Watanabe Akaike (or: widely applicable) information criterion. The difference of the expected log pointwise predictive density (ELDP, i.e. the expected predictive accuracy of the model) to the best model is calculated additionally, together with the standard error of this difference.
