# Supplementary material for: Decomposing the role of alpha oscillations during brain maturation
Source: eLife. 2022 Aug 25;11:e77571. doi: 10.7554/eLife.77571 (PMC9410707; doi:10.7554/eLife.77571)
Supplement: Supplementary file 2. — A: Bayesian regression model results of HBN subsample of subjects without any given diagnosis.B: Bayesian regression model results of full HBN sample with categorical sub-diagnosis predictor.C: Bayesian regression model results for canonical alpha power measures in the full HBN sample. [file elife-77571-supp2.docx]

**Supplementary statistics tables of the main dataset**

**A**

*Bayesian regression model results of HBN subsample of subjects without any given diagnosis*

|  | β_predictor_ [CI] | |
| --- | --- | --- |
| Outcome | age | gender |
| Alpha peak frequency | 0.40 [0.22 0.58] | -0.11 [-0.31 0.08] |
| Total individualized alpha power | -0.21 [-0.40 -0.03] | -0.38 [-0.57 -0.21] |
| Relative individualized alpha power | 0.10 [-0.10 0.30] | -0.25 [-0.46 -0.06] |
| Aperiodic-adjusted individualized alpha power | 0.21 [0.02 0.41] | -0.32 [-0.53 -0.13] |
| Aperiodic intercept | -0.42 [-0.59 -0.25] | -0.37 [-0.55 -0.21] |
| Aperiodic slope | -0.38 [-0.55 -0.20] | -0.33 [-0.51 -0.17] |

*Note:* CI = 99.17% Credible Interval, gender variable is coded as: 1=female, 0=male.

**B**

*Bayesian regression model results of full HBN sample with categorical sub-diagnosis predictor*

|  | β_predictor_ [CI] | | | | | |
| --- | --- | --- | --- | --- | --- | --- |
| Outcome | age | gender | diagnosis: ADHD (inattentive) | diagnosis: ADHD (combined) | diagnosis: Other | age*gender |
| alpha peak frequency | 0.41 [0.33 0.49] | -0.09 [-0.15 -0.03] | -0.07 [-0.18 0.03] | -0.08 [-0.19 0.03] | -0.07 [-0.18 0.04] | -0.02 [-0.14 0.10] |
| total individualized alpha power | -0.31 [-0.39 -0.24] | -0.36 [-0.43 -0.30] | 0.02 [-0.09 0.12] | 0.01 [-0.09 0.12] | 0.01 [-0.09 0.11] | 0.13 [0.01 0.25] |
| Relative individualized alpha power | 0.14 [0.06 0.23] | -0.33 [-0.40 -0.27] | -0.00 [-0.11 0.11] | -0.01 [-0.12 0.10] | 0.01 [-0.10 0.12] | -0.05 [-0.18 0.08] |
| aperiodic-adjusted individualized alpha power | 0.23 [0.15 0.30] | -0.38 [-0.44 -0.32] | -0.02 [-0.13 0.08] | -0.05 [-0.16 0.05] | -0.03 [-0.13 0.08] | -0.05 [-0.17 0.08] |
| aperiodic intercept | -0.54 [-0.62 -0.48] | -0.36 [-0.42 -0.31] | -0.01 [-0.11 0.08] | 0.00 [-0.10 0.09] | -0.02 [-0.11 0.08] | 0.08 [-0.03 0.19] |
| aperiodic slope | -0.45 [-0.52 -0.38] | -0.38 [-0.43 -0.32] | -0.04 [-0.13 0.06] | -0.03 [-0.13 0.07] | -0.03 [-0.13 0.06] | -0.05 [-0.16 0.06] |

*Note:* CI = 99.17% Credible Interval

**C**

*Bayesian regression model results for canonical alpha power measures in the full HBN sample.*

|  | β_predictor_ [CI] | | | | |
| --- | --- | --- | --- | --- | --- |
| Outcome | age | gender | diagnosis: ADHD | diagnosis: Other | age*gender |
| total canonical alpha power | -0.10 [-0.17 -0.02] | -0.39 [-0.45 -0.33] | -0.02 [-0.12 0.08] | -0.01 [-0.12 0.10] | 0.11 [-0.01 0.23] |
| Relative canonical alpha power | 0.36 [0.29 0.43] | -0.32 [-0.38 -0.26] | -0.03 [-0.13 0.06] | -0.01 [-0.11 0.09] | -0.07 [-0.18 0.05] |
| aperiodic-adjusted canonical alpha power | 0.31 [0.24 0.39] | -0.38 [-0.44 -0.32] | -0.05 [-0.15 0.04] | -0.02 [-0.13 0.08] | -0.07 [-0.18 0.04] |

*Note:* CI = 99.17% Credible Interval. Due to model convergence problems, models were multivariately estimated using either total, relative or aperiodic-adjusted canonical alpha power as dependent variable, each together with alpha peak frequency, aperiodic intercept and the aperiodic slope. Alpha peak frequency, aperiodic intercept and periodic slope were only added as outcome measures to account for correlations among periodic and aperiodic measures, but resulting parameter estimates did not change compared to analysis done on the same dataset using individualized alpha band measures (see 3.1, Table 3). Therefore, they are omitted here.
