# Supplementary material for: Decomposing the role of alpha oscillations during brain maturation
Source: eLife. 2022 Aug 25;11:e77571. doi: 10.7554/eLife.77571 (PMC9410707; doi:10.7554/eLife.77571)
Supplement: Supplementary file 3. — A: Validation study: Bayesian regression model results of subjects without any given diagnosis, using uninformative priors.B: Validation study: Bayesian regression model results using informative priors extracted from the main HBN analysis. [file elife-77571-supp3.docx]

**Supplementary statistics tables of the validation dataset**

**A**

*Validation study: Bayesian regression model results of subjects without any given diagnosis, using uninformative priors.*

|  | β_predictor_ [CI] | |
| --- | --- | --- |
| Outcome | age | gender |
| Alpha peak frequency | 0.44 [0.21 .68] | -0.12 [-0.36 0.12] |
| Total individualized alpha power | -0.60 [-0.80 -0.40] | -0.02 [-0.24 0.18] |
| Relative individualized alpha power | 0.08 [-0.19 0.34] | 0.02 [-0.25 0.29] |
| Aperiodic-adjusted individualized alpha power | 0.24 [-0.03 0.50] | -0.05 [-0.31 0.21] |
| Aperiodic intercept | -0.88 [-1.00 -0.76] | -0.06 [-0.17 0.05] |
| Aperiodic slope | -0.80 [-0.96 -0.64] | -0.05 [-0.21 0.10] |

*Note:* CI = 98.97% Credible Interval, gender variable is dummy coded: 1=female, 0=male.

**B**

*Validation study: Bayesian regression model results using informative priors extracted from the main HBN analysis.*

|  | β_predictor_ [CI] | | | |
| --- | --- | --- | --- | --- |
| Outcome | age | gender | diagnosis: ADHD | age*gender |
| Alpha peak frequency | 0.38 [0.32 .44] | -0.06 [-0.11 0.00] | -0.08 [-0.16 0.00] | -0.05 [-0.15 0.05] |
| Total individualized alpha power | -0.35 [-0.41 -0.30] | -0.38 [-0.43 -0.34] | -0.01 [-0.05 0.08] | 0.06 [-0.02 0.15] |
| Relative individualized alpha power | 0.13 [0.08 0.19] | -0.29 [-0.34 -0.24] | -0.03 [-0.10 0.04] | -0.02 [-0.11 0.07] |
| Aperiodic-adjusted individualized alpha power | 0.31 [0.25 0.36] | -0.39 [-0.44 -0.35] | -0.07 [-0.14 -0.01] | 0.00 [-0.08 0.08] |
| Aperiodic intercept | -0.60 [-0.64 -0.56] | -0.36 [-0.39 -0.32] | -0.01 [-0.06 0.03] | 0.01 [-0.05 0.08] |
| Aperiodic slope | -0.46 [-0.51 -0.41] | -0.37 [-0.42 -0.33] | -0.06 [-0.12 0.00] | -0.06 [-0.14 0.03] |

*Note:* CI = 98.97% Credible Interval, gender variable is dummy coded: 1=female, 0=male.
