# Supplementary material for: Decomposing the role of alpha oscillations during brain maturation
Source: eLife. 2022 Aug 25;11:e77571. doi: 10.7554/eLife.77571 (PMC9410707; doi:10.7554/eLife.77571)
Supplement: Supplementary file 4. — Bayesian regression model investigating the influence of global white matter integrity on aperiodic signal parameters in the HBN dataset. [file elife-77571-supp4.docx]

**Supplementary analysis on global white matter integrity extracted from DTI.**

*Bayesian regression model investigating the influence of global white matter integrity on aperiodic signal parameters in the HBN dataset.*

|  | β_predictor_ [CI] |  |  |  |
| --- | --- | --- | --- | --- |
| Outcome | global white matter | total intracranial volume | age | gender |
| Aperiodic intercept | 0.02 [-0.03 0.08] | 0.03 [-0.03 0.08] | -0.51 [-0.57 -0.45] | -0.35 [-0.41 -0.30] |
| Aperiodic slope | 0.04 [-0.01 0.10] | 0.04 [ -0.01 0.10] | -0.49 [-0.54 -0.43] | -0.39 [-0.45 -0.33] |

*Note:* CI = 95% Credible Interval. A Multivariate model was fitted for the predictor global white matter integrity on the aperiodic signal intercept and slope, while controlling for age, gender and total intracranial volume.
