# Supplementary material for: Decomposing the role of alpha oscillations during brain maturation
Source: eLife. 2022 Aug 25;11:e77571. doi: 10.7554/eLife.77571 (PMC9410707; doi:10.7554/eLife.77571)
Supplement: Peer review history

# Editor report: Editor's evaluation

**DOI:** 10.7554/eLife.77571.sa0

**Authors:** Laura Dugué

**Affiliations:** https://ror.org/05f82e368 Uni­ver­sité de Paris France

This is an important paper that is methodologically compelling to investigate changes in α oscillations with development. The manuscript usefully addresses a timely debate regarding methodological limitations when analyzing periodic independently of aperiodic activity.

# Decision letter

**DOI:** 10.7554/eLife.77571.sa1

**Affiliations:** https://ror.org/05f82e368 Uni­ver­sité de Paris France; https://ror.org/05g3dte14 Florida State University United States; https://ror.org/00hj8s172 Columbia University United States

Our editorial process produces two outputs: (i) public reviews designed to be posted alongside the preprint for the benefit of readers; (ii) feedback on the manuscript for the authors, including requests for revisions, shown below. We also include an acceptance summary that explains what the editors found interesting or important about the work.

**Decision letter after peer review:**

Thank you for submitting your article "Decomposing the role of α oscillations during brain maturation" for consideration by *eLife*. Your article has been reviewed by 3 peer reviewers, and the evaluation has been overseen by a Reviewing Editor and Barbara Shinn-Cunningham as the Senior Editor. The following individuals involved in review of your submission have agreed to reveal their identity: Wen Li (Reviewer #2); Thomas Donoghue (Reviewer #3).

The reviewers have discussed their reviews with one another, and the Reviewing Editor has drafted this to help you prepare a revised submission.

Essential revision:

(1A) The relation of this paper to previous work, in particular in regards to previous methodological issues and empirical claims relating to aperiodic activity, needs to be clarified.

(1B) Overall, the manuscript should more carefully balance raising the awareness of the aperiodic confound in power indices and alarming the field as to the validity of the extant findings. Words like "contradiction" may be overly alarming when the relative power index yields consistent results as the adjusted power index.

(1C) The key finding of this report is that periodic α power increases with age, which is a notably different result from some of the previous literature, as well as from the analyses within this paper that analyze total power in the α range. The interpretation of this finding provided in the paper is that the decrease in power is driven by the change in aperiodic activity, which is a sensible interpretation given the results. This interpretation could be further motivated with some minor additional analyses. If the effect of total α power reflects the change in the aperiodic activity, but periodic α power does not, then this implies that the total power and aperiodic measures should be highly correlated, whereas the periodic power and aperiodic parameters should be much less so. If this can be shown, it would help to demonstrate the interpretation.

(2A) Please further describe and report quality control measures of the spectral models.

(2B) It is interesting that when using the specParam approach, the α measure that is used is not the periodic peak from the model fit, but a measure of power from the aperiodic-adjusted log power spectrum. Is there a reason this was chosen instead of using the peak parameters from the model? Are the results the same if analyzed using the peak parameters?

(3) This paper can have even greater conceptual and methodological impact on the field by focusing on "relative" as opposed to "total" power. It is also helpful to clarify which aspects of aperiodic signal (intercept or slope) and to what extent contributes to the confound in the relative α power.

(4) Related to points #2 and #3, please illuminate how "distorted" the relative α power measure is in real biologically valid datasets. Simple correlations across these α measures could be examined to shed light on their relationship. As relative α power shows similar correlation with age as the aperiodic-adjusted α power, the authors may also directly compare these two power indices to quantify the difference.

(5) The correlation between aperiodic-adjusted α power and white matter integrity of the thalamocortical radiation is very interesting. Given the very strong association of the aperiodic α indices with age, it would be helpful to examine their correlation with the thalamocortical radiation. Also, given the common use of relative α power in the literature, it is useful to examine its correlation with thalamocortical radiation.

(6) Please address the limitations of the analysis relating changes in offset and exponent. It is unclear if there is a clear way to do this analysis in an interpretable way without estimating the rotation frequency across the group, which is itself a tricky problem, so if that can't be done in the context of this paper, that analysis may need to be removed.

(7) Please describe and report the Flanker analysis in the Results, including discussing the interpretations in terms of the effect size and difference in parameter values. There could also be further description of this analysis in the methods, including a brief description (or citation for) the Flanker task, and notes on whether this data is collected with or without EEG data, and if it is collected during at the same time as the resting state EEG data that is analyzed.

(8) The sample is not homogeneous or representative. While the control analysis has ruled out ADHD-related confounds, it is still important to note that in the Abstract and discuss that in the Discussion.

(9) Throughout the manuscript participants are referred to as "children." However, the sample is between 6 and 22 years (at some point it says 21; but the oldest participant is 21.9y, so 6 to 22 years should be written instead). Please rephrase with: the sample consisted of children, adolescents and young adults. Moreover, in the additional analysis with the extreme groups the older group is called "older children." Again, part of this group are in fact young adults.

(10) The abstract uses the term 'aperiodic offset', whereas the remainder of the paper uses 'aperiodic intercept'. This should be consistent.

(11) In the abstract, lines 40-42, the phrasing is unclear on what exactly the finding regarding the isolated periodic α activity is, and this could potentially be made clearer.

(12) On lines 266-267, the report of the analyses regarding the anatomical measures of the thalamus are not clearly reported. Given the organization of the methods, in which this is presented before the methods, some brief notes that, for example, the measures of the thalamic radiation are DTI measures, would be useful, since as presented it is unclear how this paragraph relates to the claim in the abstract that α power relates thalamo-cortical connectivity.

(13) On line 398, the word "reflects" seems to suggest more of a causal link than is supported by the correlational analysis. This should be edited.

(14) Line 404-406: this sentence needs to be reformulated.

(15) Line 721: The EHQ acronym does not seem to be introduced. Please define.

*Reviewer #1 (Recommendations for the authors):*

I really want to congratulate the authors. This is a great piece of research. Also, the manuscript is very clearly written. I absolutely enjoyed reading it.

I only have one tiny suggestion that may be addressed. Throughout the manuscript participants are referred to as "children". However, the sample is between 6 and 22 years (at some point it says 21; but the oldest participant is 21.9y, so I would write 6 to 22 years). I would say the sample consisted of children, adolescents and young adults. Moreover, in the additional analysis with the extreme groups the older group is called "older children". Again, part of this group are in fact young adults.

*Reviewer #2 (Recommendations for the authors):*As for suggestions, I think this paper can have even greater conceptual and methodological impact on the field by focusing on "relative" as opposed to "total" power. It is also helpful to clarify which aspects of aperiodic signal (intercept or slope) and to what extent contributes to the confound in the relative α power. Relatedly, it is important to illuminate how "distorted" the relative α power measure is in real biologically valid datasets. I would suggest that simple correlations across these α measures be examined to shed light on their relationship. As relative α power shows similar correlation with age as the aperiodic-adjusted α power, the authors may also directly compare these two power indices to quantify the difference. Overall, I think it is important to be very careful about the balance between raising the awareness of the aperiodic confound in power indices and overly alarming the field as to the validity of the extant findings. Words like "contradiction" may be a bit overly alarming when the relative power index yields consistent results as the adjusted power index.The correlation between aperiodic-adjusted α power and white matter integrity of the thalamocortical radiation is very interesting. Given the very strong association of the aperiodic α indices with age, it would be helpful to examine their correlation with the thalamocortical radiation. Also, given the common use of relative α power in the literature, it is useful to examine its correlation with thalamocortical radiation.

The sample is not homogeneous or representative. While the control analysis has ruled out ADHD-related confounds, it is still important to note that in the Abstract and discuss that in the Discussion.

*Reviewer #3 (Recommendations for the authors):*

My main suggestions for this paper are to:

(1) Clarify the relation of this paper to previous work, in particular in regards to previous methodological issues and empirical claims relating to aperiodic activity.

(2) Describe and report quality control measures of the spectral models.

(3) Address the limitations of the analysis relating changes in offset and exponent. I'm not sure if there is a clear way to do this analysis in an interpretable way without estimating the rotation frequency across the group, which is itself a tricky problem, so if that can't be done in the context of this paper, that analysis may need to be dropped.

(4) Describe and report the Flanker analysis in the Results, including discussing the interpretations in terms of the effect size and difference in parameter values. There could also be further description of this analysis in the methods, including a brief description (or citation for) the Flanker task, and notes on whether this data is collected with or without EEG data, and if it is collected during at the same time as the resting state EEG data that is analyzed.

# Reply: Author response

**DOI:** 10.7554/eLife.77571.sa2

Essential revision:

Reviewer #1 (Recommendations for the authors):

I really want to congratulate the authors. This is a great piece of research. Also, the manuscript is very clearly written. I absolutely enjoyed reading it.

I only have one tiny suggestion that may be addressed. Throughout the manuscript participants are referred to as "children". However, the sample is between 6 and 22 years (at some point it says 21; but the oldest participant is 21.9y, so I would write 6 to 22 years). I would say the sample consisted of children, adolescents and young adults. Moreover, in the additional analysis with the extreme groups the older group is called "older children". Again, part of this group are in fact young adults.

We thank the reviewer for pointing out this phrasing issue. When referring to the study population, we rephrased the term “children” to “children, adolescents, and young adults” and the term “older children” to “young adults”. We have adapted this throughout the manuscript and updated Figure 2 accordingly. Additionally, we corrected the sample description of the age range for the HBN sample to “5 to 22 years” and for the validation sample to “6 to 22 years”.

Reviewer #2 (Recommendations for the authors):

As for suggestions, I think this paper can have even greater conceptual and methodological impact on the field by focusing on "relative" as opposed to "total" power. It is also helpful to clarify which aspects of aperiodic signal (intercept or slope) and to what extent contributes to the confound in the relative α power. Relatedly, it is important to illuminate how "distorted" the relative α power measure is in real biologically valid datasets. I would suggest that simple correlations across these α measures be examined to shed light on their relationship. As relative α power shows similar correlation with age as the aperiodic-adjusted α power, the authors may also directly compare these two power indices to quantify the difference.

We thank the reviewer for this important comment and the helpful suggestions. We agree that focusing more strongly on relative α power, which is a commonly used measure of α activity, can have greater impact on the field.

While theoretical considerations have questioned previous results in the relative α band power, the main finding when comparing aperiodic-adjusted and relative α power is that relative α power, similar to the aperiodic-adjusted α power, indicates an age-related increase with continuing brain maturation.

As suggested by the reviewer, we have conducted correlational analyses between aperiodic-adjusted and relative α power. The results revealed that both measures are highly correlated (*r* = 0.88). However, this finding also indicates that 22.6% of the variation in aperiodic-adjusted α power is not explained by relative α power. In our analyses on the association between α power and brain maturation, the measure of relative α power yielded false negative results: Although we observed a significant age-related increase in relative α power in the full HBN sample (final included N = 1770), there were no significant effects in the HBN sample without any given diagnosis (N = 190) or in the validation sample (N = 310). In contrast, aperiodic-adjusted α power consistently showed significant age-related increases across these samples. Furthermore, correlations with age are considerably higher in adjusted α power (*r* = 0.21) than in relative α power (*r* = 0.11). This leads to the conclusion that the two indices of α power are in fact related and that confounding factors in the computation of relative α power (as shown in the simulations in Appendix 1 – Figure 1) may weaken its association with age and can therefore cause null findings. Therefore, we argue that aperiodic-adjusted α power should be preferred for the analysis of brain maturation.

As also suggested, we further conducted correlation analyses between all major indices: total, relative, and aperiodic-adjusted α power and aperiodic intercept and slope. These analyses showed very similar correlations between relative α power and both aperiodic signal components (*r*_intercept_ = 0.34, *r*_slope_ = 0.35), indicating no distinctive confounding effects of either the aperiodic intercept or the aperiodic slope. The correlations between aperiodic-adjusted α power and aperiodic signal components (*r_i_*_ntercept_ = 0.34, *r*_slope_ = 0.36) are of very similar magnitude to that of relative α power.

Importantly, total α power showed considerably larger correlations with the aperiodic signal (*r*_intercept_ = 0.84, *r*_slope_ = 0.65). As suggested by reviewer #3 (comment #5), this motivates the interpretation that whereas total α power results are likely driven by changes in the aperiodic signal, the age-related increase in both aperiodic-adjusted and relative α power indicate the true oscillatory changes in the α band.

The correlational analyses are summarized in (Author response table 1):

**Author response table 1.** Pearson correlation coefficients between the different measures of α power, aperiodic intercept and slope and age.

	Total α power	Relative α power	Aperiodic- adjusted α power	Aperiodic intercept	Aperiodic slope
Total α power		0.66	0.64	0.84	0.64
Relative α power			0.88	0.34	0.35
Aperiodic- adjusted α power				0.34	0.36
Aperiodic intercept					0.89

To further highlight the comparison of relative and aperiodic-adjusted α power, we included a detailed report of the results on relative α power in the analysis of thalamic anatomical measures, in which relative α power showed a similar positive association to that of aperiodic-adjusted α power (see also answer #3 to reviewer #2) and thus provided additional evidence for the validity of relative α power. Adding relative α power to the post hoc analysis of the relation between α power measures and flanker task performance showed similar results, indicating that both relative and aperiodic-adjusted α power are significantly associated with better performance in this visual-attentional task.

We carefully edited the manuscript to set more focus on the relative α power by adapting the abstract and extending the methods and Results sections by describing these new findings on thalamic anatomical measures and Flanker task scores. We further extended the Discussion section “Relative vs. aperiodic-adjusted α power” and the conclusions section in the discussion.

Abstract:

“First, the well-documented age-related decrease in total α power was replicated. However, when controlling for the aperiodic signal component, our findings provided strong evidence for an age-related increase in the aperiodic-adjusted α power. As reported in previous studies, relative α power also indicated a maturational increase, yet the effect is of considerably smaller magnitude, thus indicating an underestimation of the underlying relationship between periodic α power and brain maturation. This underestimation might be explained by the fact that changes in other frequency bands and in the aperiodic signal can potentially affect the measures of relative α power as shown by simulation studies.”

Results (2.1):

“To investigate the inherent associations between the different measures of α power and aperiodic activity, we further conducted a post hoc correlational analysis. This analysis aimed to illuminate the differences and similarities between the three measures of α power and the potential confounding effects of the aperiodic signal. The two aperiodic indices, intercept and slope, were highly interlinked, and both exhibited high correlations with total individualized α power. At the same time, both aperiodic-adjusted and relative individualized α power indicated a considerably weaker association with the aperiodic indices. Furthermore, whereas relative and aperiodic-adjusted α power were highly related, this association was weaker between relative and total α power and aperiodic-adjusted and total α power. Table 2 summarizes the result of the correlational analysis.

Results (2.3):

“Both the left and right thalamic radiation showed significant associations with aperiodic-adjusted individualized α power, which did not reach significance level with total individualized α power. Relative α power showed similar results to those observed in aperiodic-adjusted α power.

Results (2.4):

“Both relative and aperiodic-adjusted individualized α power showed a significant positive association with task performance, while the effects of age and gender were controlled for. […]

General discussion:

“The aperiodic-adjusted individualized α power increased significantly from childhood to adolescence, which is consistent with the results obtained from relative α power in the present study and in previous literature (Clarke et al., 2001; Cragg et al., 2011; Díaz de León et al., 1988; Harmony et al., 1995; John et al., 1980; Somsen et al., 1997). The aperiodic signal showed a decreased intercept during brain maturation and a flattened slope. Results were largely consistent across the subsample of the HBN dataset without any given diagnosis, the full HBN dataset, and the validation analyses. However, in the validation dataset and the HBN subsample without any given diagnoses, aperiodic-adjusted but not relative α power showed significant age-related increases, indicating a risk of false-negative results when investigating relative α power in brain maturation.

[…]

Importantly, when relating α power measures to anatomical measures derived from DTI, only aperiodic-adjusted and relative, but not total α power showed a significant relation to the white matter integrity of the thalamic radiations”

Discussion “Relative vs. aperiodic-adjusted individualized α power”:

“Post hoc simulations indicate that changes in power in other frequency bands (see Appendix 1 – Figure 1A) induce changes in relative α power even when true oscillatory α power is kept constant. Furthermore, changes in the aperiodic signal induce a confound in the relative α power measure (see Appendix 1 – Figure 1B). This is further supported by simulations performed by Donoghue et al. (2020a) and Donoghue et al. (2021). Consequently, the increase in relative α power observed with increasing age needs to be interpreted with caution, as changes in other frequency bands and in the aperiodic signal can potentially bias this finding. Our study confirmed an age-related decrease of the aperiodic intercept and a flattening of the aperiodic slope. Hence, because these changes in the aperiodic signal could induce changes in relative α power even though the true oscillatory pattern remains stable, this relative measure is no conclusive indicator of a true age-related increase in α power.

[…]

Importantly, the aperiodic-adjusted individualized α power showed consistent significant age-related increases in the main HBN sample, the HBN subsample of children without any given diagnosis, and the validation dataset. Conversely, the relative individualized α power only showed a significant association with age in the largest main HBN sample. Therefore, our results indicate that there is a risk of false negative results when investigating relative α power changes from childhood to young adulthood in sample sizes commonly used in neurophysiological studies. Hence, the developmental increase on periodic α power may be underestimated when using relative α power indices, which might be explained by a potential confounding bias of the aperiodic signal components and power in other frequency bands on the relative α power (see supplementary simulation studies in Appendix 1). Overall, aperiodic-adjusted α power should be preferred over relative α power when analyzing developmental trajectories during brain maturation.”

Discussion “Conclusions”:

“Furthermore, the current report provides partial support of previous literature on age-related increases in relative α power, as these effects could only be replicated in the large dataset, but not in the smaller samples. Consequently, aperiodic-adjusted α power should be preferred over relative α power, as the latter measure underestimated age-related changes of true periodic α power and therefore yielded a risk of false negative results.”

Overall, I think it is important to be very careful about the balance between raising the awareness of the aperiodic confound in power indices and overly alarming the field as to the validity of the extant findings. Words like "contradiction" may be a bit overly alarming when the relative power index yields consistent results as the adjusted power index.

We thank the reviewer for pointing out the issue of possibly overly alarming phrases.

We carefully edited these phrases throughout the manuscript and removed words such as “contradiction.”

Abstract:

Original: “Consequently, earlier interpretations on age related changes of α power need to be fundamentally reconsidered, incorporating changes in the aperiodic signal”

Rephrased: “Consequently, earlier interpretations on age-related changes of total α power need to be reconsidered, as elimination of active synapses rather links to decreases in the aperiodic intercept.”

Discussion “Age-related increase in aperiodic-adjusted individualized α power”:

Original: “Previous studies on age-related changes of α power during brain maturation speculated that decreased total oscillatory power may be due to synaptic pruning processes (e.g., Cragg et al., 2011) and thus reflect decreased spiking activity. The increase in aperiodic-adjusted α power contradicts these interpretations, decomposing the neural power spectra rather indicates that these processes relate to changes in the aperiodic signals intercept (see below).”

Rephrased: “Previous studies on age-related changes of α power during brain maturation speculated that decreased total oscillatory power may be due to synaptic pruning processes (e.g., Cragg et al., 2011) and thus reflect decreased spiking activity. The increase in aperiodic-adjusted α power provides new insights into these interpretations: Decomposing the neural power spectra rather indicates that these processes relate to changes in the aperiodic signals intercept (see Discussion section “Maturational changes in aperiodic signal components”).”

Discussion “Conclusions”:

Original: “Accounting for these confounding factors, and using the largest openly available pediatric sample, the present report demonstrates that the age effect on aperiodic-adjusted individualized α power shows the opposite direction as earlier assumed when investigating total α power.”

Rephrased: “Accounting for these confounding factors, and using the largest openly available pediatric sample, the present report demonstrates that aperiodic-adjusted α power increases during brain maturation”

The correlation between aperiodic-adjusted α power and white matter integrity of the thalamocortical radiation is very interesting. Given the very strong association of the aperiodic α indices with age, it would be helpful to examine their correlation with the thalamocortical radiation. Also, given the common use of relative α power in the literature, it is useful to examine its correlation with thalamocortical radiation.

We thank the reviewer for this thoughtful comment. Indeed, we investigated the relationship between the aperiodic signal components and white matter integrity of the thalamic radiation as part of a post hoc analysis (lines 457-461 and supplementary file 4 in the initial submission), but did not find any significant associations. To improve accessibility for the reader, we decided to integrate this analysis into the analyses of the main manuscript.

We further agree that investigating the relationship between relative α power and the white matter integrity of the thalamic radiation is relevant, given its previous application in developmental research. Therefore, we added the relative α power to the analysis investigating the relationship with the thalamocortical radiation*.* These results show that relative α power shows a similar relation to the thalamocortical radiation as that of aperiodic-adjusted α power.

Subsequently, we updated the corresponding method and result section and expanded the Discussion section.

Method section (4.3.4):

“The dependent variables for Models 4, 5, and 6 were total individualized α power, aperiodic-adjusted individualized α power, relative individualized α power and the aperiodic intercept and slope.

[…]

For the five outcome variables, the resulting significance level was 0.0148, yielding 98.52% credible intervals.”

Result section (2.3):

“Both the left and right thalamic radiation showed significant associations with aperiodic-adjusted individualized α power, which did not reach significance level with total individualized α power. Relative α power showed similar results as observed with aperiodic-adjusted α power. No significant associations were found between the aperiodic intercept and slope and the thalamic radiations.

General discussion:

“Importantly, when relating α power measures to anatomical measures derived from DTI, only aperiodic-adjusted and relative α power showed a significant relation to the white matter integrity of the thalamic radiations, but total α power did not.”

Discussion “Relative vs. aperiodic-adjusted individualized α power”:

“Additionally, the analyses relating α power measures to possible neuroanatomical (thalamocortical connectivity) and behavioral (visual attention task performance) correlates yielded very similar significant positive associations with both relative and aperiodic-adjusted α power.”

Discussion “Maturational changes in aperiodic signal components”:

“The flattening of the aperiodic signal in this age range may also be reflected in the commonly observed age-related decrease of power in low frequencies accompanied by an increase in power in higher frequencies (Cragg et al., 2011; Whitford et al., 2007). This phenomenon was speculated to be related to the elimination of synapses or changes in white matter structure (Segalowitz et al., 2010; Whitford et al., 2007); however, no significant relation of the aperiodic slope with white matter integrity of the thalamic radiation was found in the analyses performed here. An additional post hoc analysis also indicated no relation between the aperiodic signal parameters and global white matter integrity (see supplementary file 4).”

The sample is not homogeneous or representative. While the control analysis has ruled out ADHD-related confounds, it is still important to note that in the Abstract and discuss that in the Discussion.

We thank the reviewer for this comment and agree that this information is important and valuable for the broad readership of the journal. We therefore adapted the Abstract and expanded the limitation section in the Discussion.

Abstract:

“Using multivariate Bayesian generalized linear mixed models, we examined aperiodic and periodic parameters of α activity in the largest openly available pediatric dataset (N = 2529, age range 5-22 years) and replicated these findings in a preregistered analysis of an independent validation sample (N = 369, age range 6–22 years). Both datasets included typically developing controls and participants diagnosed with psychiatric disorders.”

Discussion “Limitations”:

“A limitation of the present study is the composition of the samples investigated, as they contain a large proportion of children, adolescents, and young adults in whom psychiatric disorders were diagnosed. Consequently, the samples are not representative of the general population in this age range. This may present a confound to the analysis of age trajectories of α power and the aperiodic signal, because psychiatric disorders have previously been linked to differences in resting state EEG band power (for a comprehensive review, see Newson and Thiagarajan, 2018) and the aperiodic slope (e.g., Robertson et al., 2019). However, control analyses using only healthy subsamples showed very similar results to analyses of the full sample. Additionally, the main and the validation analysis controlled for possible confounding effects by adding a categorical diagnosis variable as an additional predictor. No significant associations were found between clinical diagnoses and either oscillatory or aperiodic signal components within either dataset.”

Reviewer #3 (Recommendations for the authors):

My main suggestions for this paper are to:

(1) Clarify the relation of this paper to previous work, in particular in regards to previous methodological issues and empirical claims relating to aperiodic activity.

We thank the reviewer for this elaborate and helpful comment on the contextualization of our work.

We acknowledge that the suggested simulations of previous work (Donoghue, T., Dominguez, J., and Voytek, B., 2020; Donoghue, T., Schaworonkow, N., and Voytek, B., 2021) provide important findings on possible confounds in relative band power measures and in fact made similar points as our supplementary simulation examples. Therefore, the new version of the manuscript provides suitable citations to this previous work in the introduction section (lines 98-103) and put less emphasis on the supplementary simulation studies performed in the present report:

“Additionally, non-oscillatory changes in the power spectrum introduce confounds in the analysis of relative band power measures (see simulated example in Appendix 1 – Figure 1B). This was previously observed by simulations showing that non-oscillatory changes affect both relative band power measures (Donoghue, Schaworonkow, and Voytek, 2021) and band power ratio measures (Donoghue, Dominguez, and Voytek, 2020a).”

Because the simulations performed in Donoghue, T., Schaworonkow, N., and Voytek, B. (2021) also provide evidence for confounding effects of oscillatory peak frequencies in power measures, we further cited this work when introducing the problem of fixed frequency band analysis in brain maturation (lines 79-86):

“A potential confound is the utilization of fixed-frequency boundaries (e.g., 8–13 Hz), which neglects the slowing of the IAF during development. For instance, peak frequency in childhood is around 6 Hz but increases to 10 Hz in adolescents. Hence, age-related power decreases are underestimated when the slower α oscillation of younger children is not properly captured by predefined frequency limits, which leads to lower power values. Consequently, individualized α frequency bands need to be extracted, which are centered on the individual IAF of each subject (see also Donoghue et al. (2021) for simulations visualizing confounding effects of the peak frequency on band power).“

In response to the comment that the paper understates previous work on aperiodic activity, we would like to clarify: Our statement on lacking evidence for the reported significant association between age and aperiodic signal components and aperiodic-adjusted α power referred specifically to the context of brain maturation in the age range here investigated. We further refined this statement, differentiating between evidence for maturational changes in aperiodic signal components and in aperiodic-adjusted α power. As suggested by the reviewer, we updated the corresponding paragraph including references to Donoghue et al. (2020) and Hill et al. (2022) (lines 125-140):

“Recent studies adopted this methodology and found age-related changes in the aperiodic signal (i.e., decreased intercept and flattened slope) during childhood and adolescence (Cellier, Riddle, Petersen, and Hwang, 2021; Hill, Clark, Bigelow, Lum, and Enticott, 2022) and from childhood to middle age (Donoghue et al., 2020a; He et al., 2019). These results further pointed out the importance of considering the aperiodic signal in the investigation of α power during brain maturation. However, it remains largely unknown how aperiodic-adjusted α power evolves during this critical phase of life. The few studies performed so far have not found any significant association between aperiodic-adjusted α power during childhood and adolescence (Cellier et al., 2021; Hill et al., 2022) and from childhood to middle age (He et al., 2019). Due to comparatively small sample sizes in these studies, it remains unclear whether the aperiodic-adjusted α-power truly remains stable in this period of life or whether too little statistical power was provided to detect changes in this newly emerging measure of α power. Furthermore, conventional measures of total and relative α power were either not reported (Cellier et al., 2021; Hill et al., 2022), or did not show any relation to age (He et al., 2019). Hence, comparisons and integration of these results with the large body of literature investigating maturational changes in total and relative α power remain limited.”

In addition, following the reviewer's helpful comment, we added a reference to the review of He (2014) when introducing the aperiodic signal (lines 111-121):

“The aperiodic signal contains important physiological information (see He 2014 for a comprehensive review of the functional significance and potential generative mechanisms of aperiodic activity). More specifically, the aperiodic slope has been linked to the synchronicity of activity in the underlying neural population (Miller, Sorensen, Ojemann, and den Nijs, 2009; Usher, Stemmler, and Olami, 1995) and its balance between excitatory and inhibitory activity (Gao, Peterson, and Voytek, 2017). Importantly, the aperiodic slope is modulated by task performance and sensory stimulation (e.g., He, 2014). Conversely, the aperiodic intercept has been linked to general spiking activity (Voytek and Knight, 2015). Overall, the aperiodic signal needs to be considered during the analysis of spectral power rather than measuring power relative to the absolute zero (e.g., Donoghue et al., 2020b).”

(2) Describe and report quality control measures of the spectral models.

We thank the reviewer for pointing out the lack of reporting quality control measures from the specParam models. Indeed, we had applied the criteria as described in the preregistration to the analyses of both the main HBN dataset and the validation dataset, but these were not described and reported in the main manuscript. We updated the corresponding method section by adding:

Methods (4.2.6):

“Data was only used for further analysis when the model fit of the specParam model was above a threshold of R^2^ >0.90.

In the analysis of the main HBN sample, an overall high specParam model fit was observed across the sample (mean R^2^ = 0.9943, sd = 0.0098). Similar model fits were observed in the validation dataset (mean R^2^ = 0.9941, sd = 0.0120). Model fit was assessed for each of the five occipital electrodes separately (see 4.2.7). Across all subjects, only 11 out of 9705 model fits were below the cut-off of R^2^ <0.90 in the HBN sample, and 6 out of 1675 in the validation sample. Consequently, in 99.94% of subjects, all five electrodes could be used to estimate the average occipital periodic and aperiodic parameters. For those subjects with insufficient model fits for specific occipital electrodes, the average occipital periodic and aperiodic parameters were calculated from the average of the remaining electrodes with adequate model fit. In the HBN sample, the numbers of occipital electrodes available in these subjects were: Two (N = 1 subject), three (N = 1 subject) and four (N = 6 subjects). In the validation sample, these numbers of available electrodes were similar: Three (N = 2 subject), four (N = 2 subjects). No subject was excluded based on the specParam model fit.”

Based on the reviewer’s comment, we further investigated the relation between the specParam model fit and age, gender, and diagnoses (no diagnoses, ADHD, other diagnosis), controlling for recording site and handedness (EHI) in the full HBN dataset:

specParammodelfit∼age+gender+diagnosis+EHI+site

Significant associations were found for age (b = -0.0002, p = 6.63e-10) and gender (b female = -0.0015, p = 7.73e-15). To rule out possible confounds in the analysis of α power and aperiodic signal components, we ran an additional control analysis, adding a predictor labeled “specParam model fit” to the main statistical analysis (eq. 2, section 2.2.8):

$$\left[dv^{\prime }s\right]∼age*gender+diagnosis+EHI+site+specParam\;model\;fit$$

Adding the model fit as an additional predictor, the new statistical analyses showed significant positive associations of the specParam model fit with the magnitude of the α power measures and the aperiodic signal parameters. However, the original results and conclusions remained unaffected.

As the preregistered analysis does not allow any modifications and analysis should be consistent across the main and the validation datasets, we did not change our main statistical models but added the analysis described above as supplementary control analysis and referenced them in the Method section.

Methods (4.2.6):

“A series of control analyses were conducted: The first control analyses indicated a small but significant relation between age and gender with the specParam model fit (see Appendix 4). Controlling for this in the statistical model by adding the specParam model fit as an additional predictor did not change any of the main results (see Appendix 4 – Table 1).”

Appendix 4:

“Control analyses were performed to investigate the relationship between the specParam model fit and age, gender and diagnoses (no diagnoses, ADHD, other diagnosis) controlling for recording site and handedness (EHI) in the full HBN dataset. A linear model was formulated as

specParammodelfit∼age+gender+diagnosis+EHI+site

No significant effects were found in diagnosis, EHI or site; however, age (b = -0.0002, p = 6.63e-10) and gender (b female = -0.0015, p = 7.73e-15) showed significant negative associations with the model fit.

To rule out possible confounding effects of the specParam model fit in the analysis of α power and aperiodic signal components, additional control analyses were performed, adding a predictor labeled “specParam model fit” to the main statistical analysis (eq. 2, section 2.2.8). Therefore, for the main HBN dataset, a multivariate Bayesian regression model (brms) was defined as

[dv′s]∼age∗gender+diagnosis+EHI+site+specParammodelfit

As summarized in Appendix 4 – Table 1, a significant association of the specParam model fit with the magnitude of the α power measures and the aperiodic signal parameters was observed in the main HBN dataset. However, the original results and conclusions remained unaffected: A significant negative effect of age was found on the aperiodic intercept and slope and on total individualized α power. Aperiodic-adjusted and relative individualized α power and the α peak frequency showed a positive significant association with age. All of these six dependent variables showed a significant association with gender (i.e., smaller values for females as compared to males) and no significant associations with the clinical diagnosis.”

We acknowledge that the cut-off defined a priori for exclusion of bad specParam model fits (R-squared < 0.9) was an arbitrary decision, which was based on visual inspection of model fits in previous analyses of data of healthy young and old adults. Additionally, the default specParam fitting parameters applied here possibly resulted in model overfitting (mean R-squared = 0.9943, sd = 0.0098 in the full HBN sample).

To rule out possible resulting confounding effects, we ran additional control analyses following the procedure described in the guidelines by Ostlund et al. (2022). We randomly subsampled 10% of the HBN dataset. First, we applied the default specParam fitting parameters as previously in the initial submission (peak width limits: [0.5 12], max number of peaks: infinite, minimum peak height: 0, peak threshold: 2 sd above mean, aperiodic mode: fixed). Subsequently, the proportion of model underfits and overfits were determined using mean absolute error (MAE) >0.1 for underfitting and MAE < 0.025 for overfitting as cutt-offs. The definitions of these MAE cut-offs were adopted from the example analysis in Ostlund et al. (2022), who investigated a sample of children (mean age = 9.97, sd = 0.95). Applying these cut-offs yielded significant loss of data in the subsample analyzed (5.3% overfitting, 0.001% underfitting). Consequently, to minimize overfitting, we changed the model fit parameters as suggested in Ostlund et al. (2022): peak width limits: [1 8]; max number of peaks: 6; minimum peak height: 0.1; peak threshold: 2 sd above mean; aperiodic mode: fixed. Applying specParam again to data of the same 10% subsample of the HBN data yielded very little loss of data (0.001% underfitting, 0.4% overfitting).

Subsequently, all analyses described in 4.2.3 to 4.2.8 were repeated for the full HBN dataset while applying these updated specParam fitting parameters. With these parameter settings, 0.2% of all fitted specParam models were underfitting, and 0.5% were overfitting in the full sample, applying the new exclusion criteria (MAE > 0.1 or MAE < 0.025). The control analysis showed results highly consistent with the previous analysis reported in Table 1 in section 2.1.

Overall, although the specParam fitting parameters and exclusion criteria (R^2^<0.9) applied in the main analysis may possibly result in an overfitting of the specParam models, these parameters turned out applicable in the context of analyzing aperiodic and periodic α signal components during brain maturation. As the new exclusion criteria would interfere with the definitions in the preregistered analysis (R^2^ < 0.9), we also added these new control analyses in the supplementary and added a reference in the Method section.

Methods (4.2.6):

“Additional control analyses were subsequently performed to investigate whether possible overfitting of the specParam models (mean R^2^ = 0.9943, see above) confounded the results. Following the guidelines of Ostlund et al. (2022), specParam model fitting parameters and data exclusion criteria were adapted to minimize both overfitting and underfitting of the model (for the details about this approach see Appendix 5). Results were highly consistent with the main results reported in Table 1 and did not indicate any changes to the main results (see Appendix 5 – Table 1).”

Appendix 5:

“To rule out potential resulting confounding effects of specParam model overfitting (mean R-squared = 0.9943, sd = 0.0098 in the full HBN sample) and the cut-off defined a priori for exclusion of bad specParam model fits (R^2^ < 0.9), additional control analyses were performed following the procedure described in the guidelines by Ostlund et al. (2022). Following these guidelines, we randomly subsampled 10% of the HBN dataset. First, we applied the default specParam fitting parameters as previously in the initial submission (peak width limits: [0.5 12], max number of peaks: infinite, minimum peak height: 0, peak threshold: 2 sd above mean, aperiodic mode: fixed). Subsequently, the proportion of model underfits and overfits were determined using the cut-offs mean absolute error (MAE) >0.1 for underfitting and MAE < 0.025 for overfitting. The definitions of these MAE cut-offs were adopted from the example analysis in Ostlund et al. (2022), who investigated a sample of children (mean age = 9.97, sd = 0.95). Applying these cut-offs yielded significant loss of data in the subsample analyzed (5.3% overfitting, 0.001% underfitting). Consequently, to minimize overfitting, the model fit parameters were changes as suggested in Ostlund et al. (2022): peak width limits: [1 8]; max number of peaks: 6; minimum peak height: 0.1; peak threshold: 2 sd above mean; aperiodic mode: fixed. Applying specParam again to data of the same 10% subsample of the HBN data yielded very little loss of data (0.001% underfitting, 0.4% overfitting).

Subsequently, all analyses described in 4.2.3 to 4.2.8 were repeated for the full HBN dataset, applying these updated specParam fitting parameters. With these parameter settings, 0.2% of all fitted specParam models were underfitting, and 0.5% were overfitting in the full sample, applying the new exclusion criteria (MAE > 0.1 or MAE < 0.025).

The control analysis showed highly consistent results compared to the previous analysis reported in Table 1 in section 2.1, see Appendix 5 – Table 1 for detailed results.”

(3) Address the limitations of the analysis relating changes in offset and exponent. I'm not sure if there is a clear way to do this analysis in an interpretable way without estimating the rotation frequency across the group, which is itself a tricky problem, so if that can't be done in the context of this paper, that analysis may need to be dropped.

We thank the reviewer for this valuable contribution. Following this comment, we tested how strongly the conclusion of our analyses are affected by changing the rotation frequencies. Figure 2c in the main manuscript visualizes the aperiodic signal for the 20% youngest and the 20% oldest participants. We agree with the reviewer that this figure indicates a larger rotation frequency than the 19Hz used initially, therefore we reran our analysis using increasingly large rotation frequencies from 19 Hz up to 40 Hz. While the bootstrap analysis still provides strong evidence for an age-related decrease of the aperiodic intercept at 25 Hz (99.98% of 10.000 bootstraps show smaller intercept than expected by the mere rotation of the aperiodic signal), this evidence decreases with increasing frequency (35 Hz: 63% of the bootstraps show smaller intercept than expected by rotation; 40 Hz: only 18% of the bootstraps show smaller intercept than expected by rotation). The reviewer correctly pointed out that estimating the true rotation frequency is a non-trivial problem, especially considering interindividual variance in the rotation frequency. Thus, we decided to drop this analysis from the manuscript. Instead, we discussed this issue in the Discussion section.

Discussion “Maturational changes in aperiodic signal components”:

“An alternative explanation for the finding of a decreased intercept needs to be considered: As pointed out by He et al. (2019), a maturational flattening of the aperiodic signal imposes a decrease in the intercept due to the high correlation between the aperiodic intercept and slope (also observed in the main HBN dataset, r = 0.89, see Table 2). To estimate whether the observed decrease of the aperiodic intercept is larger than expected by the rotation of the aperiodic slope requires estimation of not only the decrease of the aperiodic slope, but also the frequency at which the aperiodic signal rotates. Future research is needed to provide means by which this estimation can be achieved, considering also interindividual differences in the rotation frequency.”

(4) Describe and report the Flanker analysis in the Results, including discussing the interpretations in terms of the effect size and difference in parameter values. There could also be further description of this analysis in the methods, including a brief description (or citation for) the Flanker task, and notes on whether this data is collected with or without EEG data, and if it is collected during at the same time as the resting state EEG data that is analyzed.

We thank the reviewer for this comment. In the initial submission, the continuous variables (Flanker score, age, total α power, aperiodic-adjusted α power) were not standardized; therefore, the magnitude of the regression coefficients were neither interpretable nor comparable. We adjusted this oversight by z-transforming the continuous variables and reran the analysis, adding relative α power as a dependent variable, in line with reviewer #1’s recommendations. In the new analysis of the association between aperiodic-adjusted α power and performance in the Flanker task, the Flanker score showed a standardized β of 0.073, which was highly significant (*p* = 0.001) while controlling for the effect of age. Thus, we are confident that the relationship between aperiodic-adjusted α power and Flanker task performance is of sufficient magnitude to be interpreted, indicating an effect size of almost one third of the magnitude of the very prominent age effect; the effect of age in this model was 0.233 (p <0.001). The effect of Flanker task performance on total α power was considerably smaller, showing a drop of effect size magnitude by a factor of 1.7 (standardized β = 0.043) compared to the magnitude of the effect on adjusted α power. This effect also fails to reach significance when adjusting the significance level for multiple comparisons using the Nyholt correction (adjusted significance level = 0.0264, p = 0.047). Additionally, based on the valuable comment #1 of reviewer #2 on the importance of focusing on the findings in relative α power, we added this measure in the analysis of Flanker task performance (see also answer #1 to reviewer #1). Results indicate similar effects of Flanker task performance on relative α power (standardized β = 0.068, *p* = 0.002) as on aperiodic-adjusted α power. However, we note that the comparison between the different measures of α power was not the focus of this analysis, as preceding analyses had indicated that aperiodic-adjusted α power should be preferred over total α power when investigating brain maturation.

In the new version of the manuscript, we restructured the presentation of the Flanker task analysis, providing more details in the method section (4.5), moving the results from the supplement to the Results section (2.4) and updating the corresponding Discussion section:

Methods (4.5):

“Post hoc analyses were performed to investigate the relationship between the different measures of α power and attentional performance. The Flanker task of the National Institutes of Health Toolbox Cognition Battery (Gershon et al., 2013) was employed as a measure of attentional performance. In each of the 40 trials of this task, a set of stimuli is presented to participants, who are asked to indicate the direction (left or right) of the central stimulus. These stimuli are either arrows, for participants of the age 8 year or older, or fish, for children younger than 8 years. Therefore, participants need to focus attention on the central stimulus and suppress information from surrounding stimuli, which may be congruent or incongruent with the direction of the central stimulus. Thus, the task aims to measure inhibitory control and visual selective attention. This data was collected as part of the HBN study. Participants performed a computerized version of the task in a separate assessment without neurophysiological recording. The score was calculated based on a combined measure of accuracy across trials and reaction time (Zelazo et al., 2014). Age standardized scores were automatically extracted by the NIH test software (for more details, see National Institutes of Health and Northwestern University.: http://www.healthmeasures.net/2-uncategorised/209-nih-toolbox-technical-manuals-for-ac). The final sample size, for which both task data and EEG were available, was N = 1757 (age range, 5-22 years, mean age = 10.81, sd = 3.44).

The score in the Flanker task was used as the predictor in the linear models. The univariate linear models controlled for age, gender, and handedness (EHI) and were defined as:

α power ∼ Flanker score + age + gender + EHI

All predictors and outcome variables were standardized (z-transformed). The models were fitted separately for three outcome variables: total individualized α power, relative individualized α power, and aperiodic-adjusted individualized α power. To account for multiple comparisons with three different measures of α power, the effective number of tests was calculated using Nyholt’s approach, and Šidák correction was applied to adjust the significance level of 0.05 (Nyholt, 2004), yielding a corrected significance level of 0.0264.”

Results (2.4):

“Post hoc analyses were performed to investigate the relation between the different measures of α power and performance in a visual spatial attention task: the Flanker task of the National Institutes of Health Toolbox Cognition Battery (Gershon et al., 2013). Both relative and aperiodic-adjusted individualized α power showed a significant positive association with task performance while controlling for effects of age and gender. The effect of Flanker task performance on total α power showed a considerably smaller effect size (standardized β dropped by a factor of 1.7 compared to aperiodic-adjusted α power) and failed to reach significance level when adjusting for multiple comparisons. Table 5 summarizes the results of the linear models.”

Discussion “Age-related increase in aperiodic-adjusted individualized α power”:

“In fact, post hoc analyses supported this hypothesis by providing evidence that oscillatory α power is linked to performance in visual attention tasks, assessed by performance in the Flanker task. While age, gender and handedness were controlled for, relative and aperiodic-adjusted individualized α power showed significant positive relations to the attentional performance when adjusting for multiple statistical comparisons, but total individualized α power did not.”
